# Bio-Based Polymers with Antimicrobial Properties towards Sustainable Development

**DOI:** 10.3390/ma12040641

**Published:** 2019-02-20

**Authors:** Alexandra Muñoz-Bonilla, Coro Echeverria, Águeda Sonseca, Marina P. Arrieta, Marta Fernández-García

**Affiliations:** 1 Instituto de Ciencia y Tecnología de Polímeros (ICTP-CSIC), C/Juan de la Cierva 3, 28006 Madrid, Spain; sbonilla@ictp.csic.es (A.M.-B.); cecheverria@ictp.csic.es (C.E.); agueda@ictp.csic.es (Á.S.); 2 Facultad de Ciencias Químicas, Universidad Complutense de Madrid (UCM), Av. Complutense s/n, Ciudad Universitaria, 28040 Madrid, Spain; marina.arrieta@gmail.com

**Keywords:** bio-based polymers, antimicrobial, biodegradable, sustainable, eco-friendly

## Abstract

This article concisely reviews the most recent contributions to the development of sustainable bio-based polymers with antimicrobial properties. This is because some of the main problems that humanity faces, nowadays and in the future, are climate change and bacterial multi-resistance. Therefore, scientists are trying to provide solutions to these problems. In an attempt to organize these antimicrobial sustainable materials, we have classified them into the main families; i.e., polysaccharides, proteins/polypeptides, polyesters, and polyurethanes. The review then summarizes the most recent antimicrobial aspects of these sustainable materials with antimicrobial performance considering their main potential applications in the biomedical field and in the food industry. Furthermore, their use in other fields, such as water purification and coating technology, is also described. Finally, some concluding remarks will point out the promise of this theme.

## 1. Introduction

Nowadays, plastics have gone from being outstanding materials that make life easier for us to being a serious concern for our ecological system. The European Council has pointed out the need to reduce our dependency on fuel and gas imports and to create sustainable energy, that is, achieve sustainable development by 2030. The 17 goals that cover this sustainable development include food security, health, sustainable consumption and production, the sustainable management of natural resources, clean oceans, and climate change [1]. Bio-based polymers have emerged as a potent solution for replacing petroleum-based polymeric materials and reducing the dependence on the depleting crude oil reserve. Besides this, many of the existing bio-based polymers can be biodegradable; in particular, natural bio-based polymers, such as polysaccharides and proteins, but also several synthetic biopolymers, such as poly(lactic acid). Biodegradability is also an important and desired property in many applications, including food packaging and agricultural applications, and contributes to sustainability as it reduces the waste impact of oil-based polymers. Nowadays, although the bio-plastics market represents only about 1% of the 335 million tons of plastic that the world produces annually [2,3], their production is continuously growing [4]. In some of the uses of biopolymers, additional properties are also needed; for instance, antimicrobial properties are desired in food packaging and biomedical devices, wherein microbial contamination can cause serious problems for public health and safety.

On account of this background, in this article we intend to show the capacities of bio-based polymers to be antimicrobial materials, centered on both natural and synthetic polymers.

There are extensive and excellent reviews about antimicrobial polymeric materials [5,6,7,8,9,10,11,12] in which the methodologies of encapsulation and blending with antimicrobial organic and inorganic compounds as well as their possible mechanisms of action are discussed [9]. However, most of them are mainly focused on fossil-oil derivatives. On the other hand, there are also many reviews about bio-based polymers [13,14,15,16,17,18,19,20]; however, only a few are related to antimicrobial activity [21,22]. Therefore, this review does not intend to gather all of the works performed to date but give hints on the subject and make the general public aware of the great possibilities of sustainable polymeric materials.

First, we will mention polysaccharides, which are the most abundant and exploited family. Following the natural systems, the proteins with antimicrobial activity will be described. Then, synthetic systems based on natural products will be analyzed; specifically, polyesters and polyurethanes. Since the literature regarding natural and bio-based antimicrobial polymeric materials is significantly wide, we focus the analysis mainly on the research performed in the field during recent years. It is not our purpose to do an extensive review; instead, we will highlight some of these interesting materials. Finally, we will conclude with some reflections on this hot topic.

## 2. Polysaccharides

Polysaccharides are the macromolecules that belong to the components of life, together with proteins and nucleic acids. They determine the functionality and specificity of species. Their functionalities divide them into structural, storage, and gel-forming polysaccharides. Due to their abundance and excellent properties, such as biodegradability, they are unique materials to develop interesting antimicrobial bio-based materials.

### 2.1. Chitosan

**Figure 1:**
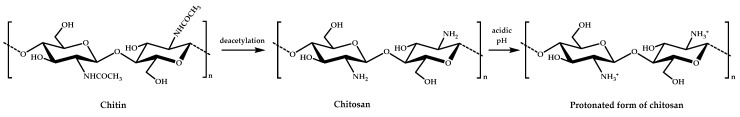
The chemical structure of chitin and chitosan and the protonated form of chitosan.

Chitosan (CS) is a linear polysaccharide with inherent antimicrobial activity that is derived from naturally occurring chitin, which is, after cellulose, the most common biopolymer on earth. It is sourced mainly from crustacean shellfish and certain fungi. Chitosan is a partially or completely *N*-deacetylated derivative of chitin, chemically composed of *N*-acetylglucosamine and glucosamine units joined through β(1−4)glycosidic linkages, and has primary amino groups that provide a positive charge under acidic pH (pK_a_ about 6.3) and decent antimicrobial properties against a wide range of micro-organisms (Figure 1) [23,24].

Although the exact mechanism of action is still not completely understood, the most accepted mechanism is based on electrostatic interactions between positively charged chitosan and the negatively charged micro-organism membrane [25]. Nevertheless, other modes of action, such as interactions with DNA or the formation of complexes with metal ions, seem to be involved [26]. This antimicrobial activity is strongly affected by its structural characteristics, such as molecular weight or degree of deacetylation, and by environmental conditions, such as pH, temperature, or ionic strength [27]. Compared with other antimicrobial polymers, chitosan offers several advantages, as it has a natural origin, is biodegradable, biocompatible, and nontoxic for mammalian cells, and has been approved by the U.S. Federal Drug Administration (FDA) and the E.U. as safe (GRAS, Generally Recognized As Safe) for tissue engineering, drug delivery, wound dressing, dietary use, and plant protection applications. Besides this, chitosan has excellent film-forming ability and good mechanical and barrier properties; thus, it has great potential in food packaging [21]. However, its biocidal activity and solubility are reduced in neutral pH conditions [28], which limit its use in many applications. Therefore, chemical modifications of chitosan, typically either at amino (the secondary C2 NH_2_ group) or hydroxyl groups (the primary C6 OH and secondary C3 OH groups), aim to produce derivatives with enhanced properties to widen its applications [29,30]. A huge number of studies have been carried out on the preparation of antimicrobial chitosan derivatives mainly via quaternization and carboxylation. However, all of these modifications propose to improve its solubility and antimicrobial activity while also maintaining its original biodegradability and biosafety. Next, the most common and recent functional groups and derivatives used to improve its antimicrobial activity without affecting its inherent properties are discussed viz. by chemical modification and blending with organic and inorganic antimicrobial agents.

#### 2.1.1. Chitosan Modification

Probably the most common method for introducing a permanent positive charge into chitosan chains is by the formation of quaternary ammonium groups by either direct quaternization of the primary amino group at the C2 position or by incorporating such groups at any of the reactive moieties (hydroxyl and amino groups). For instance, in a recent study, chitosan derivatives with triple quaternary ammonium groups were synthesized via Schiff-based reactions. Although the resulting samples with a high positive charge exhibit significantly enhanced antifungal activity, the preparation method required multiple steps [31].

In another study, chitosan derivatives were prepared by reaction with different quaternary ammonium salts containing a bromide end-group capable of reacting with the amino or hydroxyl groups of chitosan [32]. The ammonium salts benzalkonium bromide, pyridinium bromide, and triethyl ammonium bromide were previously obtained by a quaternization reaction between 1,4-dibromobutane and the respective tertiary amines. These chitosan derivatives with quaternary ammonium groups showed much lower minimum inhibitory concentration (MIC) values against Gram-negative *Escherichia coli* and Gram-positive *Staphylococcus aureus* bacteria than neat chitosan. Also, in the case of *S. aureus*, the type of substitution influences the activity, with better properties for the pyridinium derivative. Although an important improvement of the activity is generally obtained [33], these chemical modifications often lead to unselective reactions at the amine, the hydroxyl, or both, as occurred in the last example. For instance, the *N*-methylation with methyl iodide typically provokes partial *O*-methylation [34]. Similarly, chitosan derivatives only modified at the OH positions are exceptional. Besides this, it is difficult to obtain a high degree of substitution in most of the cases, in particular with long alkyl chains, as these syntheses normally need to be carried out in acidic conditions or heterogeneous media [35].

Recent studies have been directed at obtaining better selectivity and a high degree of substitution by using several protecting groups. Sahariah et al. [36,37] have developed an efficient method for the selective modification of chitosan with up to 100% substitution of the amino groups. They prepared protected di-tertbutyldimethylsilyl (TBDMS) chitosan and introduced quaternary ammonium groups with different alkyl chain lengths by reductive amination. All of the prepared derivatives showed bactericidal properties and good selectivity when tested with human red blood cells (RBCs). It was also shown that the activity was influenced by the length of the alkyl chain and by the tested micro-organisms; derivatives with a short alkyl chain presented high activity against *S. aureus*, while longer alkyl chains were more active against Gram-negative *E. coli* and *Enterococcus faecalis* bacteria [36]. These derivatives also demonstrated effectiveness towards *S. aureus* biofilms, especially those with short alkyl chains [37].

In another recent work, the quaternary ammonium groups were introduced exclusively at the hydroxyl groups by previous protection of the –NH_2_ groups via a Schiff-based condensation reaction with benzaldehyde [38]. By this way, it is possible to prepare positively charged chitosan derivatives with free primary amino groups, which is important as these amino groups have a key role in the biological activity of chitosan, such as in its antioxidant activity. The obtained *O*-quaternized chitosans showed an improved water solubility and antibacterial activity against Gram-positive bacteria. Remarkably, the cytotoxicity for the AT2 cell line was significantly lower than that of the free quaternary ammonium salts.

A different strategy was followed in a recent work, in which quaternized chitosan samples with alkyl chains were prepared by an external acid-free method [39]. In this approach, a quaternary ammonium molecule containing carboxylic acid was synthesized, which acted as a reactant for attachment onto the amino groups of chitosan via 1-ethyl-3-(3-dimethylaminopropyl) carbodiimide/*N*-hydroxysuccinimide (EDC/NHS) chemistry and also acted as an acid for the dissolution of chitosan. The antimicrobial activity was tested against planktonic Gram-positive *Staphylococcus epidermidis* and *E. coli* bacteria, and *Candida albicans* fungi, by evaluating the MIC and minimum bactericidal and fungicidal concentration (MBC and MFC, respectively) and against biofilms. Although the chitosan derivative showed growth inhibition and biocidal effects, the results against Gram-negative bacteria were modest.

The preparation of carboxyalkyl chitosan derivatives, especially carboxymethyl chitosan (CMC) polymers, is also a common strategy to improve the water solubility of chitosan and enhance the antimicrobial activity over the whole range of pH [40]. Typically, carboxymethyl chitosan is synthesized through carboxymethylation of the primary amino and alcohol groups, leading to *N*-CMC and *O*-CMC chitosan, respectively, as well as *N,N*-CMC and *N,O*-CMC derivatives. The incorporation of the carboxymethyl group at the reactive positions of chitosan (amine and hydroxyl) can be controlled by the reaction conditions, such as temperature or concentration [41,42,43]. In general, the antimicrobial activity of *O*-CMC is greater in comparison with the rest of the derivatives, as it presents a higher number of free amino groups [41], although other parameters, such as the degree of deacetylation, the degree of substitution, the molecular weight, and the pH of the medium, can affect its antimicrobial capacity [44].

In addition, carboxyalkyl chitosan is commonly modified by the introduction of other functional groups in order to improve the activity. For instance, several thiosemicarbazone *O*-carboxymethyl CS derivatives have been prepared towards a condensation reaction of thiosemicarbazide *O*-carboxymethyl CS with *o*-hydroxybenzaldehyde, *p*-methoxybenzaldehyde, and *p*- chlorobenzaldehyde [45]. The antimicrobial activity of the prepared derivatives was tested against Gram-positive *Bacillus subtilis* and *S. aureus*, Gram-negative *E. coli* bacteria, and *Aspergillus fumigatus*, *Geotrichum candidum*, and *C. albicans* fungi using the inhibition zone method. The microbiological results showed that both the antibacterial and antifungal activities of the thiosemicarbazone *O*-carboxymethyl chitosan derivatives were better than those of the original *O*-carboxymethyl chitosan, especially the chloro-derivative.

In recent years, the effectiveness of carboxyalkyl chitosan against biofilm formation has also been studied [46,47]. A carboxymethyl chitosan with an *O*-carboxymethylation degree of ~90% was tested against Gram-positive and Gram-negative bacterial biofilm formation, and the results indicated that CMC provoked a reduction of 74.6% at 2.500 mg/mL and 81.6% at 0.156 mg/mL, respectively [46]. CMC also demonstrated the capability to prevent bacterial biofilm formation in dynamic conditions. Although the mechanism of action was not fully understood, it seems that the presence of CMC induces the flocculation of bacteria by surface charge neutralization that prevents initial bacterial adherence and cell–cell interaction. This group has also demonstrated the efficacy of CMC on the inhibition of a fungal biofilm of *Candida tropicalis*, *Candida parapsilosis*, *Candida krusei*, and *Candida glabrata* [47].

Chitosan and chitosan derivatives have also been conjugated with several cationic amino acids and antimicrobial peptides with the purpose of improving their activity. The incorporation of amino acids, such as arginine [25,48], typically leads to derivatives with excellent antimicrobial properties and high water solubility. Arginine has a guanidine group with a pKa of ~12.5; thus, it is positively charged over almost the whole range of pH. Arginine can be easily attached onto chitosan by using, for example, an EDC/NHS coupling reaction between the amino group of chitosan and the carboxylic acid of arginine [48]. Guanidine molecules have been also attached onto chitosan [49,50,51]; however, their selective introduction requires more complex strategies, such as the use of protecting groups [52].

Although the coupling of amino acids onto CS has been extensively explored, in the last few years the incorporation of antimicrobial peptides has attracted more attention. Antimicrobial peptides (AMPs), both host defense peptides and their synthetic analogues, are promising candidates as antimicrobial agents due to their high efficiency and low probability to induce bacterial resistance [53,54,55]. Furthermore, the preparation of these cationic peptide-polysaccharides might enhance the selectivity towards bacteria in comparison with mammalian cells, as their structure mimics the peptidoglycans found in the bacterial membrane [56]. Cationic chitosan-*graft*-polylysine and chitosan-*graft*-poly(lysine-*ran*-phenylalanine) have been prepared by *N*-carboxyanhydride (NCA) ring-opening copolymerization of α-amino acids initiated from the amino groups of chitosan. The resulting derivatives showed outstanding broad spectrum antimicrobial properties against Gram-negative *Pseudomonas aeruginosa* and *E. coli* and Gram-positive *S. aureus* bacteria (MIC values between 5 and 20 μg/mL) and the fungi *C. albicans* and *Fusarium solani* (MIC values between 0.2 and 0.9 μM,) while maintaining very high selectivity over human RBCs. In another strategy, the antimicrobial peptide poly(lysine_11_-*stat*-phenylalanine_10_), prepared by NCA ring-opening copolymerization, was modified by hexamethylene diisocyanate and then statistically grafted onto the acid-functionalized chitosan [57]. In this approach, the residual −COOH groups had to be further esterified, as an important decrease in the antibacterial activity was observed that partially counteracted the positive charge.

**Figure 2:**
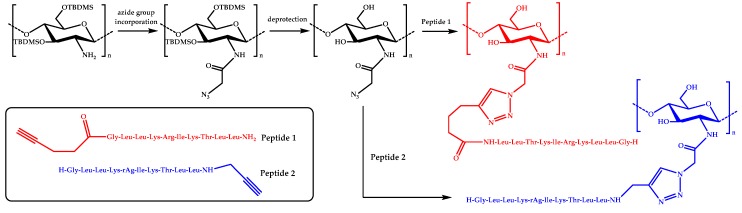
A schematic representation of the preparation of anoplin-chitosan derivatives by CuAAC click chemistry.

In more recent studies, click chemistry has been used to couple AMPs onto chitosan. For instance, a potent antimicrobial peptide, Dhvar-5 (sequence LLLFLLKKRKKRKY), with an *N*-terminal propargylglycine was attached onto azide-functionalized CS via Cu(I)-catalyzed azide-alkyne cycloaddition (CuAAC) [58]. Similarly, an anoplin peptide was grafted onto chitosan by using a CuAAC coupling reaction [59]. Azido moieties were anchored onto the amine groups of chitosan by using *tert*-butyldimethylsilyl (TBDMS) protection, whereas the anoplin peptides were synthesized bearing either N– or C–terminal alkyne groups (Figure 2).

Then, several conjugates were obtained by varying the content of the attached peptide. Some of the resulting derivatives exhibited enhanced antimicrobial activity against *S. aureus*, *E. faecalis*, *E. coli*, and *P. aeruginosa* bacteria compared to anoplin or chitosan. In particular, the conjugates were very effective against *E. coli*, with MIC values as low as 4 μg/mL. More importantly, the hemotoxicity was significantly reduced in the case of the anoplin-chitosan derivatives.

Other click chemistry reactions, such as thiol-ene click chemistry, have been employed to prepare peptide–chitosan conjugates. The cationic peptide ε-poly(l-lysine) was attached onto chitosan in order to prepare broad-spectrum antimicrobial compounds, as the ε-poly(l-lysine) is effective against bacteria but presents poor activity against fungi [60]. In this coupling reaction, the chitosan was first functionalized with methacrylate groups, and the terminal amino group of the peptide was thiolated with homocysteine thiolactone hydrochloride. After the click reaction, the obtained cationic peptide-polysaccharides demonstrated both antibacterial and antifungal activities. In addition, the conjugates showed low hemolytic activity, good in-vitro biocompatibility when tested with bone mesenchymal stem cells, and scant evidence of in vivo toxicity.

**Figure 3:**
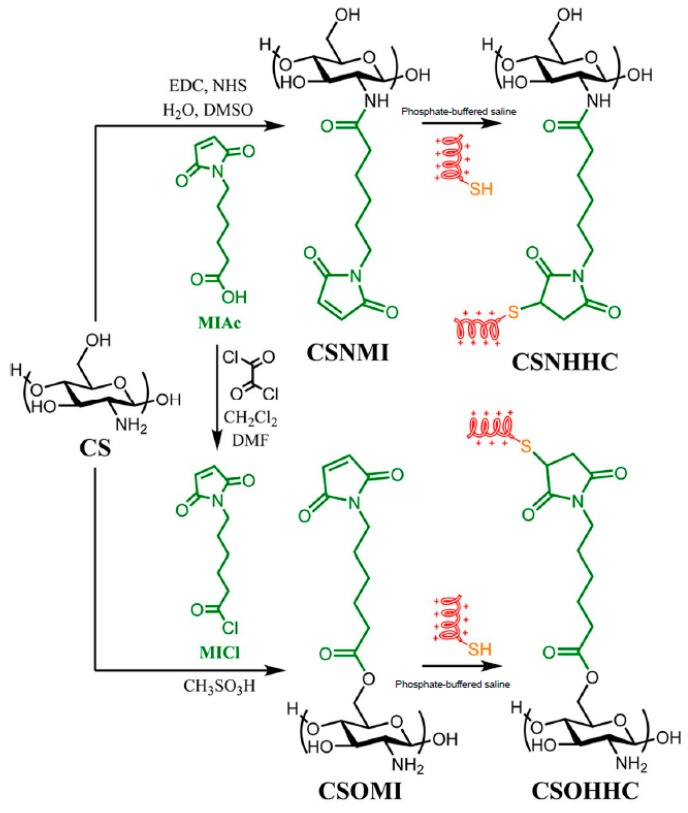
Preparation of the peptido-polysaccharides by conjugation of the HHC10 antimicrobial peptide (AMP) to the C-2 (amine) or C-6 (hydroxyl) positions of chitosan. Adapted from [62].

In another recent example, cysteine-terminated HHC10 (KRWWKWIRW) AMP was grafted onto the C2 (amino) or C6 (hydroxyl) reactive centers of CS by thiol-maleimide click conjugation [61] (Figure 3). Remarkably, the peptide-polysaccharide with free amino groups of chitosan backbone (CSO-HHC) displayed higher antibacterial activity than the corresponding conjugate with the modified amino groups (CSN-HHC) due to its capacity for protonation, which increases its water solubility and the positive charge. Likewise, both conjugates showed lower hemolytic activity and cytotoxicity than the free peptide due to the effect of chitosan.

#### 2.1.2. Chitosan Mixed with Antimicrobial Organic Compounds

Essential oils (EOs) are a product of aromatic plants, which contain multiple substances, including terpenes and aromatic and aliphatic compounds, such as esters, ethers, aldehydes, ketones, lactones, and phenols [62,63]. They have received great attention during the last few years due to their antioxidant and antimicrobial activities, in particular in the food industry [64]. However, the direct use of essential oils for food preservation is often limited due to their cost, poor solubility, toxicity, and aroma, which may impact on the sensory perception of foods. In this sense, essential oils can be incorporated into films, coatings, or capsules in a reduced dose that maintains their efficacy. Much effort has been made in the development of chitosan materials with essential oils, as chitosan has great potential as an active ingredient as well as in bio-based packaging films and edible films [65]. In general, the incorporation of essential oils improves the effectiveness of chitosan against fungi and food-borne bacteria. Essential oils can be incorporated either directly in the formulation of chitosan films [66] or previously encapsulated [67]. Likewise, the preparation of micro- and nanocapsules of chitosan derivatives as food additives has been extensively explored [68] as well as the covalent attachment of some components, such as gallic acid, onto chitosan [69,70]. For instance, in a recent publication, a rosemary essential oil was incorporated onto chitosan-montmorillonite nanocomposite films in different amounts (0.5%, 1%, and 2% v/v) [71]. Films containing the essential oil exhibited antimicrobial activity on the contact surface for both Gram-positive and Gram-negative bacteria tested by the inhibition zone method, whereas the chitosan films did not present any activity. Results in growth media obtained by the colony forming units (CFU) method, indicated, however, that the presence of the essential oil did not affect the antibacterial activity, and the montmorillonite decreased the activity as it can interact with chitosan and phenolic compounds of the rosemary essential oil. Thus, in the preparation of composite films for a food packaging application, the components and additives that are added to improve the mechanical and physico-chemical properties could affect their antimicrobial properties, so an optimal design is normally required. In another example, nanoemulsions of carvacrol were incorporated onto carboxymethyl chitosan films that were previously obtained by electrospray from CMC microgels [72]. The resulting composite films showed good antibacterial activity against *S. aureus* and *E. coli* and also the capability to prolong the shelf-life of wheat bread.

In the last few years, the nanoencapsulation of essential oils has attracted more attention compared to microencapsulation as smaller particles improve the solubility and dispersibility of the compounds. A clove essential oil was encapsulated by chitosan nanoparticles via the emulsion ionic gelation technique [73]. The resulting loaded nanoparticles demonstrated enhanced fungal activity against *Aspergillus niger* in comparison with empty chitosan nanoparticles and free oil. Similar results were found for a rosemary essential oil nanoencapsulated in chitosan/γ-polyglutamic acid nanoparticles, with a significant increase in the antibacterial activity against *B. subtilis* [74]. Likewise, an essential oil of Cyperus articulates was loaded into chitosan nanoparticles by an oil-in-water mixture and ionic gelation method [75]. These loaded particles also showed lower MIC values against *S. aureus* and *E. coli* compared to free oil and unloaded chitosan nanoparticles. However, these nanoparticles exhibited a higher cytotoxicity effect against MDA-MB-231 cells, probably due to the slow release of oil components encapsulated in the chitosan nanoparticles.

#### 2.1.3. Chitosan with Metallic Nanoparticles

An important and highly explored strategy for improving the antimicrobial activity of chitosan is the incorporation of metal or metal-oxide nanoparticles (NPs), including Ag, Cu, ZnO, and TiO_2_ NPs, and the preparation of nanocomposites. Among all existing nanoparticles, silver nanoparticles (AgNPs) have attracted much attention due to their potent antimicrobial activity. Several approaches have been used to prepare chitosan/AgNP nanocomposites, including physical and chemical strategies, in which the main objective is to reduce agglomeration, which is considered to be an important factor that affects the antimicrobial efficacy in the nanocomposites. Common methods imply the in situ preparation of AgNPs by the chemical reduction of silver salts; however, the used reducing agents may exhibit toxicity and also could interact with the functional groups of chitosan. Thus, more environmental friendly methods are becoming a priority nowadays [76]. In this sense, it was demonstrated that chitosan can act as both a reducing and stabilizing agent in the synthesis of AgNPs [77]. For instance, AgNPs stabilized with chitosan were synthesized at a large scale by a green method using autoclave, in which chitosan functions as a reducing agent as well as a stabilizer [78]. It was shown that, while chitosan only can prevent the growth of *S. aureus*, AgNPs stabilized with chitosan are also able to inhibit the growth of *E. coli* bacteria. Moreover, the inhibition zone from a disk diffusion test increased with the presence of the AgNPs. This was due to the additional modes of action of AgNPs by disrupting the cell wall of bacteria via several pathways and also by the release of Ag^+^ ions, which can interact with bacterial DNA and proteins [79]. They also showed that the stabilization of chitosan reduces the cytotoxicity of AgNPs against L-929 fibroblast cells, which might be due to limited contact between the NPs and the cells and the controlled release of Ag^+^.

Nevertheless, there is a serious concern related to the possible toxicity of AgNPs for the human body; for instance, when they are used in food packaging [80,81]. A possible solution might be the immobilization of silver nanoparticles to limit the leakage and diffusion of AgNPs. For example, laponite, which is a synthetic clay with a nano-sized and layered structure, was used to immobilize silver nanoparticles in chitosan films [82]. In this approach, quaternized chitosan was used as a reducing agent for the synthesis of AgNPs embedded in laponite, and subsequently the modified laponite was mixed with chitosan to prepare films by the casting solvent evaporation technique. Remarkably, the resulting films only released about 5.6% of AgNPs, which was much lower than films without laponite (about 29.1%). In addition, the films showed low toxicity and good antimicrobial activity against *E. coli* and *S. aureus* bacteria and *A. niger* and *Penicillium citrinum* fungi, and were capable of extending the shelf-life of fresh litchi.

In addition to AgNPs, other nanoparticles, such as Cu and CuO NPs [83,84], ZnO NPs [85,86], and TiO_2_ NPs [87,88], have been used to improve the antimicrobial activity of chitosan. In the case of TiO_2_ nanoparticles, it is accepted that their antimicrobial activity is based on photocatalytic processes under UV-light irradiation that generate reactive oxygen species (ROS) [89,90]. However, recent studies have also demonstrated biocidal properties of TiO_2_ nanocomposites under visible light [88,91], which might improve their applicability since there is a low proportion of UV light in the total solar irradiance. Zhang et al. [88] prepared chitosan-TiO_2_ composites with efficient antimicrobial activities under visible light. They incorporated TiO_2_ nanoparticles into chitosan and evaluated the antimicrobial behavior against food-borne pathogenic microbes, including *E. coli*, *S. aureus*, *C. albicans*, and *A. niger*, under visible light irradiation (a 20 W daylight lamp). The films exerted high antimicrobial activity against the tested strains with 100% sterilization in 12 h. The good performance obtained under visible light irradiation was attributed to the decreased transmittance found in the visible light region, which enabled the films to have a photocatalytic antimicrobial effect.

### 2.2. Cellulose

**Figure 4:**
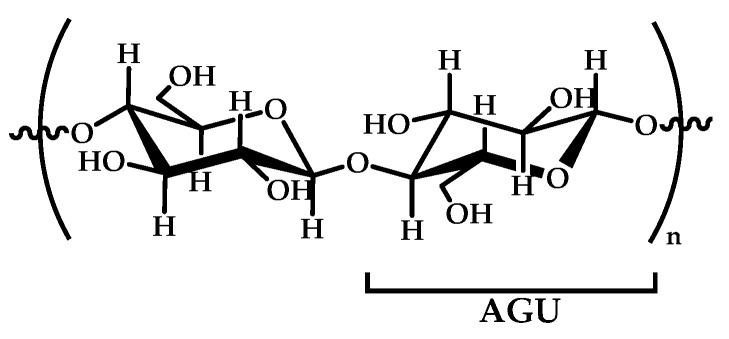
A schematic representation of cellobiose that shows the repeated anhydroglucopyranose units (AGUs).

Cellulose is a linear syndiotactic and semi-rigid homopolymer consisting of d-anhydro glucopyranose units (AGU), where each unit has three hydroxyl (OH) groups at the C2, C3, and C6 positions (see Figure 4). Cellulose is a semi-crystalline and high-molecular-weight homopolymer of β-d-glucopyranose units linked by β-1,4-linkages, where the repeat unit is a dimer of glucose: cellobiose. Cellulose and its derivatives are one of the most abundant natural biopolymers, and much progress has been made towards their study, characterization, and applications. Most cellulose derivatives are commercially available. Some are water-soluble, biodegradable, electro-neutral, and biocompatible. They are used in many industrial applications, such as packaging and textile production; however, in recent years, cellulose-based materials have been investigated regarding new advanced applications, such as sensors, liquid crystal polymers, soft-actuators, and biomaterials [92,93,94]. The increased interest in this natural polymer, obtained from renewable biomass feedstock, responds to the urgent need for the replacement of synthetic polymers to reduce the actual global dependence on fossil fuel sources, making possible the development of sustainable and ecofriendly functional materials.

As stated in the introduction, there is also an actual challenge involving the development of antimicrobial materials, which includes the development of biopolymers (natural polymers) and bio-based polymers with antimicrobial properties. Indeed, there is a deep concern regarding the increased resistance of microbes (bacteria, fungi) against actual antimicrobial agents. Micro-organisms are everywhere and they require only moisture, a source of carbon, and mild temperatures to multiply and prosper. Unfortunately, cellulose and its derivatives are an excellent medium that can serve as a supplier of moisture and even the growth of micro-organisms. So, modifications need to be done to impart antimicrobial properties to this natural polymer [95]. To do so, and also in the case of polysaccharides, three main approaches have been followed: cellulose and cellulose derivative modification (functionalization or grafting); blending with cationic molecules, essential oils, or antimicrobial polymers; and the incorporation of antimicrobial metal nanoparticles (silver, gold, etc.). Special recognition of nanocellulose is given below.

#### 2.2.1. Cellulose Modification

The first strategy followed for the development of sustainable antimicrobial cellulosic materials considers the chemical modification/functionalization or grafting onto cellulose derivative surfaces [96] so that non-leachable materials are obtained. This strategy is often applied for the modification of nanocellulose, or uses microfibrillated cellulose as starting material, as we will describe in the following section. However, based on the literature from the last three years, research related to cellulose derivatives is scarce. As an example of this approach, Wu et al. reported the preparation of nisin-grafted cellulose membranes [97]. The authors first oxidized native cellulose using sulfuric acid, and then bonded nisin amino groups onto aldehyde groups of the oxidized cellulose. The obtained nisin-grafted cellulose membranes were then tested against Gram-variable *Alicyclobacillus acidoterrestris* bacteria, which are not pathogenic to humans but are implicated in the spoilage of fruits and cause a bad taste and flavor. The performed antimicrobial test confirmed the antimicrobial activity of the cellulosic material. Besides this, the authors determined that the antimicrobial efficacy increased as the oxidation time of the native cellulose increased.

Li et al. [98], using as starting material fully bleached eucalyptus kraft pulp fibers, took advantage of the layer-by-layer (LbL) technique to modify these fibers’ surfaces with chitosan and lignin (LS), which present both antimicrobial and antioxidant properties. The electrostatic LbL technique is a simple and versatile polymer surface modification method that builds a nanostructured multilayer onto a solid substrate surface with the desired composition and properties [99]. For the preparation of multilayer deposition onto cellulosic fibers, the authors immersed the fibers into CS for a short period of time and rinsed the surface with water to remove the excess. The same procedure was followed for the deposition of the LS layer. By repeating those steps up to four times, the authors fabricated a multilayer of alternant CS and LS layers over cellulose fibers. They evaluated their antimicrobial activity by measuring their MIC against *E. coli* by the standard broth microdilution method. For the test, fibers modified with different numbers of layers, and fibers where the outermost layer was either CS or LS, were selected. The obtained results revealed that as the content of the bilayer increased the growth inhibition degree also increased, but always when the CS was located in the outermost layer.

#### 2.2.2. Cellulose Mixed with Antimicrobial Organic Compounds

Another strategy described in the literature is the incorporation of essential oils into cellulose-based matrices imparting the antimicrobial property that cellulosic material lacks [100]. For instance, Heredia-Herrero et al. developed an antimicrobial plastic film based on the cellulose derivative ethyl cellulose (EC) [101]. This derivative comes from the substitution of some hydroxyl groups of the cellulose backbone with ethyl ether groups. EC is soluble in organic solvents but insoluble in water, non-toxic, versatile, and edible. From EC, it is possible to form tough films that not very flexible; hence, the addition of plasticizers is needed. Common plasticizers, being low-molecular-weight components, have the problem of migration from the polymer matrix, which affects the material’s performance on the desired application. However, what is even more important and relevant to this contribution is that certain plasticizers also lead to serious environmental pollution in addition to affecting human health [102]. The strategy followed by Heredia-Guerrero et al. was the combination of EC with acetoxy-polydimethylsiloxane (PDMS), which interact to form hydrogen bonds, and with a clove essential oil. The EC-based film’s antimicrobial activity was tested against, *E. coli, P. aeruginosa*, and *S. aureus* bacteria. The obtained results revealed that biofilm formation by *E. coli* was significantly inhibited due to the presence of the essential oil, with a film inhibition of 44% and 57% after 24 h and 48 h, respectively. In the case of *S. aureus*, significant inhibition occurred after 48 h, with 62% of biofilm inhibition [101]. In conclusion, the authors provided the cellulosic films with antimicrobial properties as well as improved their flexibility so that they could have potential application in food packaging.

Following the strategy of incorporating an essential oil into cellulosic matrices, Liakos et al. created antimicrobial cellulosic nanofibers by an electrospinning technique. For that, the authors used cellulose acetate (CA) as a biopolymer matrix to encapsulate different EOs within the CA fibers: cinnamon, lemongrass, peppermint, rosemary, and oregano [103,104]. CA is a biodegradable compound formed from the acetylation of cellulose. This biopolymer is amorphous, odorless, non-toxic, and water-vapor permeable, and shows excellent optical properties besides a high resistance to heat and chemicals [105]. For the encapsulation of the essential oils, the authors first dissolved a cellulosic polymer in acetone and then added the corresponding essential oil to the CA/acetone solution. The obtained CA/EO electrospun fibers showed a diameter size that ranged from 1 to 3 µm approximately. The antimicrobial properties of the CA/EO fibers were evaluated against *E. coli* bacteria and *C. albicans* fungi. The results revealed that cellulosic fibers containing 6.2% and 25% EOs were able to inhibit the growth of *E. coli* bacteria. The cellulosic material owes its enhanced effectiveness to the nanostructured morphology that is provided by the used technique (electrospinning). Cellulosic electrospun fibers have a high exposed surface area compared to cellulosic flat films, allowing the micro-organisms to more easily penetrate inside, so that they can better sense the presence of the antimicrobial agent. Despite this advantageous morphology, the antifungal activity of the CA/EO fibers was not effective. The authors concluded that the lack of activity was due to the size of *C. albicans* fungi being four times larger than *E. coli* bacteria, such that they were not able to penetrate inside the cellulosic material and thus make contact with the encapsulated essential oil [103].

#### 2.2.3. Cellulose Containing Antimicrobial Metal Nanoparticles

Tran et al. developed a novel method to prepare biocompatible antimicrobial composites from cellulose and keratin with silver nanoparticles [106]. The idea for the study came from the need to fix silver nanoparticles into a matrix so that nanoparticle agglomeration or coagulation could be hindered. Following this idea, they took advantage of a previous methodology used for the green synthesis of a cellulose and keratin antimicrobial composite [107], in which ionic liquids (ILs) were used as green solvents. In this case, they introduced silver salt into a cellulose-keratine-IL solution that was further reduced to obtain a biopolymer-based composite containing silver in either its ionic (Ag^+^) or metallic (Ag^0^) form. The antibacterial property of the obtained material was tested against *E. coli*, *S. aureus*, *E. faecalis*, and *P. aeruginosa*. To evaluate the antimicrobial activity, bacteria were grown in the presence of the composites with ionic or metallic Ag, and further measured by CFU counting compared to those for the cellulose/keratin composite and the control. As determined from the experiments, both composites exhibited excellent antibacterial activity against most of the studied bacteria; however, those with metallic silver nanoparticles showed slightly better performance compared to ionic silver. The interesting properties of the cellulose/keratin composite, together with the antimicrobial activity derived from the presence of silver nanoparticles, make this sustainable biopolymer-based material useful as a potential dressing for chronic wounds treatment.

As mentioned, micro-organisms are everywhere; for instance, in paper and the paper products that are widely used in our everyday life, from bank notes to newspapers, books, and packaging paper. The distribution of this kind of material can contribute to the contamination and spreading of infectious diseases. Taking this into consideration, Islam et al. integrated antimicrobial activity into cellulose paper by the incorporation of silver nanoparticles using a mussel-inspired strategy [108]. To do so, the authors functionalized cellulose paper with dopamine molecules. This functionalized paper was then immersed in an ammoniacal silver nitrate solution; at this stage, dopamine catechol groups reduced the silver salt and subsequently held the produced nanoparticles via strong adhesion. Finally, the authors demonstrated the successful antimicrobial activity of the AgNP-decorated cellulose paper against some highly virulent fish and shrimp pathogenic bacterial strains, such as Gram-negative *Proteus mirabilis, Vibrio parahemolyticus*, *E. faecalis*, and *Serratia marcescens*.

Dairi et al. have recently developed a cellulose-acetate-based film with antimicrobial and antioxidant properties for packaging applications [109]. The strategy consisted of the use of CA and AgNPs prepared following a biogenic synthesis mediated by plants. This is a novel ecofriendly process to obtain AgNPs in which there is no need for high temperatures, high pressures, and the production of toxic chemicals [110,111,112]. In this particular case, the process consisted of the synthesis of AgNPs into a gelatin-modified montmorillonite organoclay (OM) using a *Curcuma longa* tuber aqueous extract. The final material consisted of plasticized CA films that were obtained by a solvent casting method from a solution of CA, thymol, and modified nanoparticles. The antimicrobial property of the obtained film was tested against *E. coli*, *P. aeruginosa, Salmonella enterica,* and *S. aureus* bacteria as well as *A. niger* and *Aspergillus flavus.* The antimicrobial test for the films was performed by an agar diffusion disc against micro-organisms. The authors found that the CA films presented a low bacterial inhibition zone indicative of a moderate antimicrobial activity, which is directly related to the low content of Ag within the CA film. The activity against *E. coli* bacteria was moderate even when this bacteria strain was found to be most sensitive to AgNPs. In addition, the authors concluded that the presence of the organoclay may contribute to control over the silver release for a long-lasting antimicrobial effect.

Although silver nanoparticles present antimicrobial properties, both ionic and metallic silver nanoparticles were found to be toxic above a certain concentration as mentioned above [106]. Being so, Tran et al. [113] developed a cellulosic-based biopolymer antimicrobial composite using gold nanoparticles as antimicrobial agents instead of silver. It is well-known that gold nanoparticles exhibit high antimicrobial activity against Gram-positive and Gram-negative bacteria alongside their antiviral function [114]. As the authors indicated in their study, most of the work done in this regard used synthetic polymers as carriers or matrixes for the growth or encapsulation of Au nanoparticles. Tran et al. focused their work on the use of cellulose as a matrix. They took advantage of the methodology used for the green synthesis of the cellulose and keratin antimicrobial composite mentioned earlier [106,107]. However, in this case, the authors used two different ionic liquids as solvents of both the biopolymer matrix and the chloroauric acid to obtain a cellulose/keratin/Au NP composite with antimicrobial activity. The composite was tested against methicillin-resistant *S. aureus* (MRSA) and vancomycin-resistant *Enterococcus* (VRE). The assays demonstrated that the biopolymer composite is able to inhibit 97% and 98% of the VRE and MRSA bacteria, respectively, being the Au NPs responsible for the antibacterial effect. As toxicity is the drawback for silver nanoparticles, the authors also evaluated their biocompatibility. They evaluated the cytotoxicity of the composites using human fibroblasts. Interestingly, the results revealed that the cellulose/keratin/Au NPs composites were not cytotoxic. The authors demonstrated for the first time that any possible cytotoxicity that the gold nanoparticles may have had was removed when they were incorporated into the cellulose/keratin biopolymer matrix.

#### 2.2.4. Nano-Cellulose-Based Materials (Nanocrystalline, Nanofibrillated, and Bacterial Cellulose) with Antimicrobial Activity

Research on cellulose-based materials has increased intensively; however, cellulose has some limitations related to its functionalities. In this sense, there is a growing interest regarding new nanocellulose materials, such as nanocrystalline cellulose [115,116,117,118,119], microfibrillar/nanofibrillar cellulose, and bacterial cellulose [120,121,122,123,124]. The three-dimensional hierarchical structures that compose nanocellulose open up new opportunities for new fields and applications [125]. However, as occurred with cellulose, nanocellulose-based materials lack antimicrobial properties, so it is necessary to provide them with this activity.

Nanofibrillated cellulose, obtained by mechanical disintegration from native plant fibers, is a cellulosic derivative used as a novel packaging material [126]. However, its major drawback is its vulnerability to microbe attacks, such as from cellulose-consuming fungi, for instance [127]. It is known that most of the bacterial cell walls are negatively charged; therefore, and as mentioned above, an interesting option to develop intrinsic antimicrobial materials is the use of quaternary ammonium compounds, molecules, or polymers [128]. Indeed, it has been demonstrated that these compounds could interact electrostatically with the negatively charged bacteria cell wall, causing a disruption of the membrane and posterior death [6,129,130]. With this in mind, Littunen et al. [127] proposed the chemical incorporation of quaternary ammonium compounds into nanofibrillated cellulose (NFC) to impart an antimicrobial property. For that, the authors developed two types of cationized and nanofibrillated cellulose via redox-initiated graft copolymerization with a [2-(methacryloyloxy)ethyl]trimethylammonium chloride (DMQ) monomer to obtain nanofibrillated cellulose grafted poly[2-(methacryloyloxy)ethyl]trimethylammonium chloride (NFC-PDMQ), and by etherification with a quaternary ammonium compound (NFCQ). They evaluated the antimicrobial activity of unmodified NFC and both NFCQ and NFC-PDMQ against three potential human pathogens: *Micrococcus luteus*, *E. coli* bacteria, and *Candida oleophila* yeast. As expected, unmodified NFC did not show pathogen growth inhibition. As the authors stated, the cationized sample NFCQ showed a strong broad-spectrum antimicrobial effect at a high concentration (2000 µg/mL). In contrast, the polymer-grafted NFC-PDMQ showed moderate antibacterial activity but a strong antifungal response. In addition, NFCQ was notably more efficient against the Gram-negative than the Gram-positive bacteria, but NFC-PDMQ exhibited consistent activity. A cytotoxicity test was also performed for both systems and confirmed the lack of toxicity.

Fernandes et al. [131], inspired by the intrinsic antimicrobial property of chitosan that is imparted by the amino groups along the polymer chain, chemically modified cellulosic fibrils’ surface by grafting aminoalkyl groups. In particular, the authors chose bacterial cellulose nanofibrils as the matrix for the modification. Bacterial cellulose (BC), a high-purity cellulose that is produced mainly from the *Gluconacetobacter* genus, presents physical and mechanical properties that, together with its biocompatibility, make it interesting for biomedical applications. For the surface modification, the authors used a silane chemical grafting approach to produce BC-NH_2_ nanofibrils. After confirming the surface modification of the bacterial cellulose, they evaluated the antimicrobial activity against *S. aureus* and *E. coli* bacteria using non-functionalized BC as a reference. Aminoalkyl-functionalized BC membranes showed a significant reduction in bacterial viability after 24 h.

Nanocellulose (NC) particles have been also used as reinforcing agents to improve the mechanical and viscoelastic properties of biomaterials, since it is known that the major drawback of bio-based polymeric materials is their poor mechanical, thermal, and barrier properties compared to synthetic polymers [132]. Besides this, the chemical versatility of nanocellulose allows for its modification/functionalization so that antimicrobial agents, such as nanoparticles, can be anchored [133]. As a result, nanocellulosic particles could act as antimicrobial agents in addition to reinforcing the bio-based material [118,119,125]. For instance, Spagnol et al. [134] developed silver-functionalized cellulose nanoparticles without using organic solvents that were further incorporated into a polymer matrix. To obtain nanocrystal (NC)/AgNPs, they first obtained cellulose NCs by acid hydrolysis using HCl; the hydrolysis lasted for different periods of time so that NCs with different dimensions were obtained. In the next step, the authors functionalized the NCs’ surface with succinic anhydride (NCSA) to incorporate carboxylic groups. Then, the carboxylic groups were deprotonated by adding NCSA to a sodium bicarbonate solution to act as anchoring groups for AgNPs. In the last step, the deprotonated NCSA solution and the AgNO_3_ solution were mixed together and further purified so that NCs functionalized with AgNPs were successfully obtained. The antimicrobial activity of this NC/AgNPs system was evaluated by determining their MIC against *S. aureus*, *B. subtilis*, and *E. coli* bacteria and *C. albicans* fungi by the standard broth microdilution method. The effectiveness was dependent on the morphology of the obtained NC/AgNPs. In fact, the best results were obtained for NC/AgNPs samples with smaller sizes (between 6 and 18 nm), since larger nanoparticles had difficulty penetrating into the micro-organism cells.

In a recent study, Lizundia et al. [135] designed innovative antimicrobial bio-based films composed of cellulose nanocrystals and metallic (silver, zinc oxide, and titanium dioxide) nanoparticles that show antimicrobial activity. As mentioned above, AgNPs present antimicrobial activity but with the limitation of toxicity above a certain concentration. Zinc-oxide nanoparticles have effective antibacterial activity and good catalytic, electrical, photochemical, and optical properties [136]. For the film preparation, Lizundia et al. first synthesized an NC by sulfuric acid hydrolysis of microcrystalline cellulose, giving rise to nanorods approximately 10 nm in diameter and 170 nm in length. Next, they dispersed the respective nanoparticles (rod-like ZnO, spherical Ag_2_O, and TiO_2_) in water through sonication prior to their incorporation into the aqueous NC suspension. In the final step, the prepared NC/nanoparticles dispersions were solvent-cast to form the films by evaporation-induced self-assembly. The properties of the obtained nanocellulosic films were evaluated in terms of the effect of NC dimension, shape, and chemistry in the final composite. Interestingly, the authors confirmed that the method used for the films’ preparation was derived from the formation of liquid crystal phases with a chiral nematic (cholesteric) structure. However, the incorporation of NPs affects this cholesteric structure, as reflected by the different optical properties observed by UV. The authors evaluated the antimicrobial activity against *E. coli* and *S. aureus* bacteria and the cytotoxicity on planktonic cell cultures after being in contact with the different NC-based films, and on the cells adherent to the surface of materials to determine the number of live cells. As revealed from the results regarding *E. coli*, at 3 h of incubation time, the surviving fraction of cells adherent to the surfaces showed a significant reduction in NC/Ag_2_O followed by NC/ZnO and, to a minor extent, on NC/TiO_2_ films. A similar trend was observed for planktonic cells. In the case of *S. aureus* bacteria, the surviving fraction of cells adherent to NC-based films was significantly diminished on NC/Ag_2_O, followed again by NC/TiO_2_ and NC/ZnO. The difference was observed after the direct exposition of *S. aureus* cells to the NC-based film when no significant reduction was observed for NC/TiO_2_ films, whereas for NC/Ag_2_O and NC/ZnO films an important decrease in cell survivability was detected. After 24 h of incubation, the surviving fraction of adherent cells decreased significantly for all NC films, but differences were observed for the planktonic cells, in which relevant results were only observed with the NC/Ag_2_O and NC/TiO_2_ films. In summary, the direct exposition of *E. coli* cells to NC-based films containing Ag_2_O or ZnO nanoparticles was effective at both shorter and longer incubation times. Similar results were obtained in the case of direct exposition of *S. aureus*, although its effectiveness is more significant on adherent cells at 3 h of incubation. The activity of NC-based films was less effective against planktonic cells at either 3 h or 24 h of incubation. These successful results indicated that the incorporation of nanoparticles could provide NC films with antimicrobial activity. The authors suggested their use as biomaterials and, in particular, as sustainable biomaterials for wound-healing applications.

### 2.3. Starch

Another interesting polysaccharide is starch, which is formed by a large number of glucose units linked by glycosidic bonds. Starch is a highly hydrophilic polymer that consists of linear amylose and highly branched amylopectin. It can be obtained from different botanical sources, such as potatoes, wheat, maize (corn), rice, and cassava. Starch has numerous applications in the food area because it is abundant, cheap, biodegradable, and edible [137]. However, its mechanical performance is poor; therefore, to overcome this limitation, it is usually is blended with another biopolymer, such as chitosan. In spite of this, starch does not have inherent antimicrobial properties, so these properties also need to be conferred on it.

Starch has been chemically modified to introduce cationic groups by etherification, graft copolymerization, or a combination of both. Yang and coworkers [138,139,140,141] have extensively used this type of modification to obtain flocculants for water treatment, as starch is a low-cost and effective system. These authors also tested starch’s antimicrobial activity, which has scarcely been explored in this field. They showed that *E. coli* and *S. aureus* bacteria were almost unviable after flocculation. Cationic starch was also used in combination with starch and sodium alginate to obtain polyelectrolyte films with an antimicrobial character [142]. These films have inhibitory effects on *E. coli* and *S. aureus* that are greater against Gram-positive than against Gram-negative bacteria.

**Figure 5:**
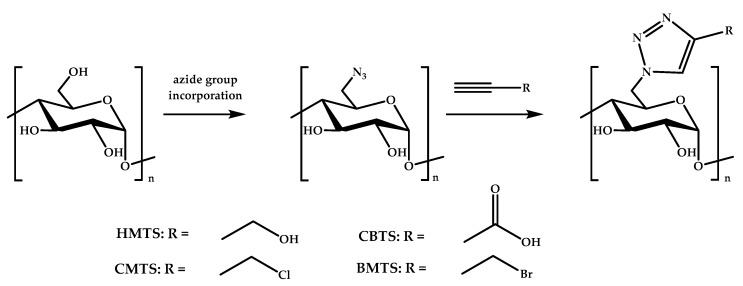
The synthesis of starch derivatives via click chemistry.

Another modification of starch performed by Guo’s group was the introduction of 1,2,3-triazole via click chemistry, which reached high yields and degrees of substitution (see Figure 5) [143]. The resulting derivatives, 6-hydroxymethyltriazole-6-deoxy starch (HMTS), 6-bromomethyltriazole-6-deoxy starch (BMTS), 6-chloromethyltriazole-6-deoxy starch (CMTS), and 6-carboxyltriazole-6-deoxy starch (CBTS), were able to inhibit the growth of *E. coli* and *S. aureus* bacteria. The best system was CBTS, followed by CMTS, BMTS, and HMTS.

This group has also incorporated quaternized phosphonium salts into starch [144]. In this work, the derivatives were tested against the common plant-threatening fungi *Watermelon fusarium*, *Phomopsis asparagi*, *Colletotrichum lagenarium*, and *Fusarium oxysporum*. The most active derivatives were those with phenyl and cyclohexyl groups. The cytotoxicity of starch derivatives was also examined against HEK-293T cells using an MTT assay. These systems presented low cytotoxicity. The cytotoxicity was higher in those systems having alkyl groups.

Indeed, one of the most common approaches to the provision of antimicrobial activity is the incorporation of antibiotics into the formulation. Microparticles formed by a polyelectrolyte complex or self-aggregation are the preferred carriers for drug administration [145,146,147]. Nevertheless, its release has to be in a controlled manner and without a toxicity effect.

As mentioned for chitosan, at present, many studies are focused on the incorporation of natural compounds [148,149]. Pattanayaiying et al. [150] have evaluated the effect of the combination of nisin (a small antimicrobial peptide approved by the European food safety authority that is used as a food preservative [134,135]) and lauric arginate^®^ (ethyl lauroyl arginate: a derivative of lauric acid, L-arginine, and ethanol) (LAE) in a thermoplastic starch/poly(butylene adipate terephthalate) film coated with gelatin against Gram-negative *Vibrio parahaemolyticus* and *Salmonella typhimurium* bacteria. This combination has a synergic effect in comparison with LAE alone, as occurred in pullulan, another polysaccharide film [151]. Recently, pouches of polyamide/low-density polyethylene were coated with blends of oxidized starch with gelatin containing LAE [152]. The authors found that the incorporation of LAE extends the shelf-life of chicken breast fillets without affecting the meat’s oxidation. Nevertheless, the release of these natural products is not always in a controlled manner. Campos-Requena et al. [153] have developed thermoplastic starch/layered silicate (TPS/LS) bionanocomposite films for the controlled release of carvacrol. This is possible due to the formation of intercalated/exfoliated structures that can tune the migration of antimicrobial carvacrol [154], which results in the increase of its half-life.

Other biocomposite films have been obtained by a combination of pea starch and guar gum containing catechins from blueberry ash and macadamia as a natural extract, as well as epigallocatechin-3-gallate from green tea [155]. These films were tested against Gram-positive *Staphylococcus lugdunensis*, *S. epidermidis*, *B. subtilis*, and *E. faecalis* bacteria, Gram-negative *Pseudomonas fluorescence*, *Klebsiella pneumoniae*, *Enterobacter aerogenes*, *S. typhimurium*, and *E. coli* bacteria, and *C. albicans*, *A. niger*, *Geotrichum candidum*, *Penicillium italicum*, *Penicillium digitatum*, *Rhizopus* sp., and *Mucor* sp. fungi. These films were able to prevent the growth of food pathogenic and spoilage micro-organisms; therefore, they can be used as edible films.

The group of Chiralt [156,157,158,159,160] has also intensively worked toward the incorporation of antimicrobial essential oils in starch-based materials for their use as preservative coatings or packaging systems. Moreover, they have also introduced proteins into starch [161], such as lactoferrin and lysozyme, as efficient antioxidant/antimicrobial systems [162,163]. In this sense, the co-encapsulation of herb extracts and lysozyme into such polysaccharides as starch, chitosan, and alginate has been demonstrated to produce more stability and durability during storage [164]. Besides this, these particles were more effective against Gram-positive *B. subtilis* and *Micrococcus luteus* bacteria and Gram-negative *E. coli* and *Serratia marcescens* bacteria. Starch was also modified with octenyl succinic anhydride for microencapsulation by electrospray, in combination with gum Arabic and nutmeg oleoresin [165]. They exhibit excellent antioxidant activity and a high retention of phenolic and flavonoid content after 60 days of storage as well as antimicrobial activity against *E. coli* and Gram-positive *Bacillus cereus* bacteria. This modification of starch was also used to stabilize emulsions of nisin and thymol (2-isopropyl-5-methylphenol) (an isomer of carvacrol) cantaloupe juice [166]. The addition of modified starch to the juice increases its capacity to retain nisin and thymol over the storage period and to inhibit the growth of Gram-positive *Listeria monocytogenes* and *Salmonella enterica* serovar Typhimurium.

Starch has been also blended with antimicrobial polymers to provide a bioactive character. Chitin nanowhiskers were added (0.5–5%) to starch, and films obtained by solvent casting were tested against *L. monocytogenes* and *E. coli* to analyze their antibacterial properties [167]. These films showed more effectiveness against Gram-positive than against Gram-negative bacteria. Moreover, they exhibited improved thermal properties and mechanical strength in comparison to native maize starch.

Additionally, starch is also extensively used as a reducing and capping agent for the synthesis of metal and metal-oxide nanoparticles, as in the case of chitosan [168,169]. Taking advantage of this ability, antimicrobial chitosan–starch–silver-nanoparticle-coated [170] cellulose papers were obtained. In a first step, starch–silver nanoparticles were synthesized, and then blended with chitosan in solution at different compositions. Afterwards, the mixture was poured onto papers and the antimicrobial activities were tested against the *E. coli* DH5α and *S. aureus* bacterial strains and the *Penicillium expansum* fungal strain. The results showed that the chitosan–starch–AgNP papers were effective against these microorganisms in comparison with papers coated with chitosan or starch–AgNPs alone, which do not present antimicrobial properties.

**Figure 6:**
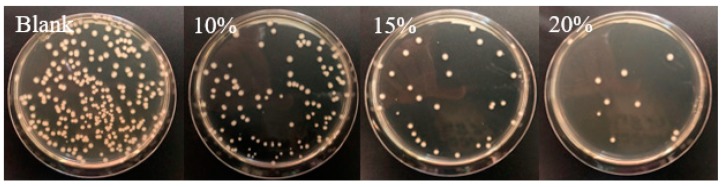
Images of the antimicrobial activities against *Escherichia coli* bacteria of coated papers with different amounts of guanidine-based starch using a shaking flask method. Reproduced from [171].

**Figure 7:**
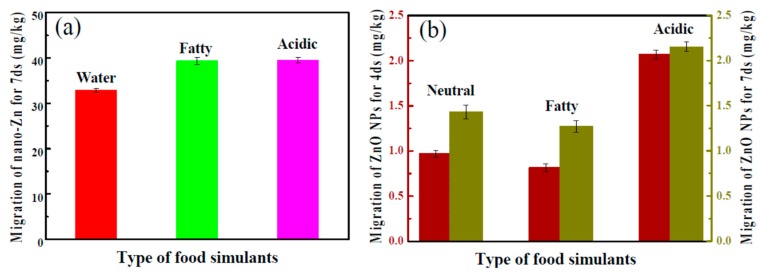
(**a**) The migration of ZnO nanoparticles (NPs) from films into different food simulants at 40 °C for 7 days; and (**b**) from coated papers for 4 and 7 days. Reproduced from [171].

In another approach, starch-based flexible coating papers with excellent hydrophobicity and antimicrobial activities were obtained [171]. These were prepared with ZnO NPs, in which carboxymethyl cellulose (CMCe) and chitosan were added to improve the compatibility between particles and matrix. The antimicrobial activity was improved with the addition of guanidine-based starch in different amounts (see Figure 6 for an illustration). Moreover, migration tests were performed in three food simulants (deionized water, 10% alcohol solution, and 3% acetic acid), according to the E.U. No. 10/2011 standard (see Figure 7). It seems clear that the migration of ZnO NPs is much higher in films than in coated papers, and, although there is migration in all of the simulants, it is within the overall migration limits prescribed by legislation.

In this sense, the group of Xiao has widely used guanidine-based systems to confer potent antimicrobial properties on cellulosic materials for their use as sanitary papers, filters, or food packaging papers [10,172,173,174].

Copper nanoparticles have also been incorporated into starch-based hydrogels to obtain antimicrobial systems [175]. These nanoparticles were synthesized in a starch medium followed by silica coating, which enhances their stability. The antibacterial activity of hydrogels with different amounts of NPs was evaluated against *E. coli* and *S. aureus,* and was maintained for at least four cycles of use. In addition, their dermal toxicity was studied, showing slight irritancy. Therefore, these hydrogels can be suitable wound-dressing materials.

Starch–graphene (G) hydrogels were obtained by Diels-Alder crosslinking reactions between furan-modified starch bismaleimide in the presence of graphene layers. These were incorporated as a conductive nanofiller to the mixture using Salvia extracts as dispersion stabilizers. The resulting hydrogels were tested against *E. coli* and *S. aureus* bacteria [176]. They present activity with low concentrations of extract, which confirms that the addition of G sheets also influences the antimicrobial efficiency. Moreover, these materials exhibit improved mechanical and conductivity performance.

### 2.4. Other Polysaccharides

In addition to the most abundant polysaccharides (starch, chitosan, and cellulose), other carbohydrate polymers, such as alginate, pectin, and κ-carrageenan, have been employed to prepare antimicrobial biopolymeric materials with high potential in a large variety of applications, especially in the food and biomedical fields [137].

Alginate is a linear anionic polysaccharide extracted from marine algae containing β-d-mannuronate and α-l-guluronate residues linked by (1,4)-glycosidic bonds. This biopolymer has found a variety of applications in biomedical science and the food industry due to its biocompatibility and gelation capability [177,178]. Several strategies have been followed in the last few years to confer an antimicrobial character on alginate-based materials. In recent investigations, sodium alginate/poly(ethylene glycol) hydrogels with antimicrobial activity were prepared by grafting the cysteine-terminated antimicrobial peptide HHC10–CYS, at different proportions, into the structure through a thiol-ene click reaction [179]. Microbiological studies of the hydrogel against *E. coli* bacteria revealed that the activity increased with the content of peptide in the hydrogel. In addition to the strong antibacterial activity, the hydrogel showed good cytocompatibility. Nevertheless, most of the works related to alginate-based materials with an antibacterial character use approaches that are mainly centered on the incorporation of antimicrobial agents into the alginate material without any chemical reaction. In fact, there is a huge number of publications on the preparation of nano- and microcapsules of alginate for the encapsulation of antimicrobial components, such as essential oils [180,181], nisin [182,183], ZnO NPs [184], and AgNPs [185]. In this respect, a common strategy is the preparation of capsules by the formation of complexes between anionic alginate and cationic polysaccharides, such as chitosan [186], or cationic peptides, such as nisin [187], with inherent antimicrobial properties. Besides this, antimicrobial films based on alginate have also been prepared by incorporating the antimicrobial agents [142,188,189,190].

Pectin is another important anionic polysaccharide rich in galacturonic acids, with a potential use in many fields, especially the food industry. Pectin is found in the cell wall of most plants; however, apple and citrus peels are almost exclusively used for the commercial production of pectin. Likewise, it presents an ability to form gels and has good gas permeability properties [137]. Typically, in pectin-based materials, the pectin is crosslinked and blended with other components to improve their physical properties and water stability. An interesting strategy to impart antimicrobial activity to pectin is the use of ions as a crosslinking agent with an antimicrobial character, such as Zn ions [191]. Similarly to alginate-based materials, pectin has been employed to prepare capsules for loading antimicrobial agents, including nisin [192] and antibiotics [193]. In the last few years, there has also been interest in the preparation of antimicrobial films based on pectin by including such agents as essential oils [194,195,196,197] and AgNPs [198].

Carrageenan has been also studied as a anionic polysaccharide material for potential applications in packaging [199]. Carrageenan is a linear sulfated polysaccharide composed of *d*-galactose and *d*-anhydrogalactose obtained from marine red algae. Among all of the carrageenan types, the κ-carrageenan type is used the most due to its good properties. A number of studies have been published in recent years related to the development of antimicrobial films based on κ-carrageenan by addition of classical AgNPs [200,201], ZnO NPs [202], CuO NPs [203], essential oils [204], and clays [205]. For instance, carrageenan-based hydrogels and dry films with antimicrobial properties were prepared by their combination with CuO and ZnO NPs [203]. Several samples were prepared, containing 1% ZnO, 1% CuO, or 0.5% ZnO/0.5% CuO, and, in general, both the mechanical and antimicrobial properties were improved with the incorporation of nanoparticles. The films showed strong antibacterial activity against *E. coli* and *L. monocytogenes*; however, an insignificant difference in the activity was observed between the different types of incorporated NPs. Equally as described in starch and cellulose, chitin nanofibrils have been used to reinforce carrageenan and to impart antibacterial properties [206]. The tensile strength and modulus of carrageenan film increased significantly with up to 5 wt% of chitin nanofibers. With respect to the antibacterial activity, the films showed high activity against *L. monocytogenes* depending on the content of chitin, but insignificant activity against *E. coli*.

## 3. Proteins/Polypeptides

Within this challenge of finding sustainable bio-based materials, natural macromolecules as proteins are playing an important role due to their versatility and it being possible to modify them enzymatically, chemically, and physically so that the desired properties can be obtained for each specific application. In general, proteins are used as additives in polymeric matrices (nisin and corn zein [207,208], soy protein [209], and wheat gluten [210]), and some examples of this can be found in the different sections of this work. However, protein-based materials have also emerged in applications as diverse as packaging [211] and biomedicine [212]. In this section, we will briefly describe the most recent work regarding sustainable antimicrobial protein-based materials for some of the most used proteins, including caseinates, keratin, and collagen.

### 3.1. Caseinates

Among animal proteins, caseinates are considered to be attractive for use in the food-processing industry; i.e., food packaging and culinary applications, since they show numerous advantageous properties, such as their natural origin, edible character, water solubility, and ability to act as emulsifiers [213,214]. Caseinates show an ability to form networks, plasticity, and elasticity, which lead to the formation of transparent films with good performance as a barrier against oxygen, carbon dioxide, and aroma compounds [213,214,215]. Sodium caseinate is more frequently used than the other caseinates, such as calcium caseinate or potassium caseinate, and, importantly, caseinates are frequently plasticized with glycerol to obtain the required flexibility for the formation and manufacture of film [213,216,217]. Moreover, caseinate-based films have attracted interest as carriers of antimicrobial substances in food-related applications [217]. The main advantage of introducing antimicrobial agents into caseinate films, as in the other described systems, is the ability to slow the diffusion of the agents through the film, allowing for its availability at a desired concentration. Therefore, smaller amounts of antimicrobial additives are needed to achieve a targeted shelf-life extension, compared with the direct addition of the antimicrobial additives onto the food surface strategy, where they quickly diffuse away from the surface, and are rapidly diluted or react with food components [216].

**Figure 8:**
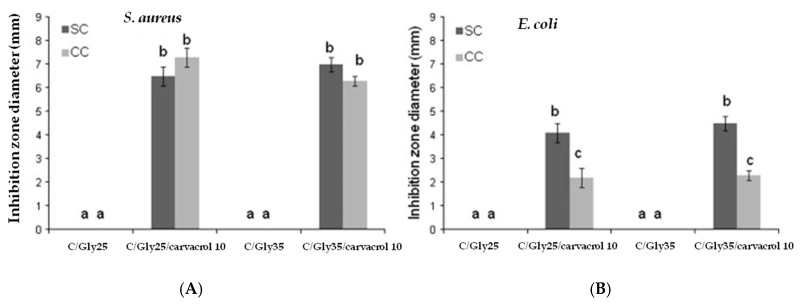
The inhibition zone (mm) observed in caseinate-based films plasticized with glycerol and loaded with carvacrol against (**A**) *S. aureus and* (**B**) *E. coli* bacteria. a–c Different letters on the bars indicate significant differences between formulations (*p* < 0.05). Reproduced from [217] with permission from Elsevier.

Noori et al. [218] have recently developed a nanoemulsion-based edible coating with strong antimicrobial activity against psychrophilic bacteria to extend the shelf-life of chicken fillets. For that, ginger (*Zingiber officinale*) EO was added to sodium caseinate matrices and the obtained films showed comparable results to the antibiotic gentamicin. Arrieta et al. added 10 wt% of carvacrol into edible matrices of both sodium and calcium caseinate, and further studied the obtained plasticized films against *S. aureus* and *E. coli* bacteria by the agar diffusion method [214,217]. Although edible films of sodium caseinate and calcium caseinate with carvacrol showed antibacterial effectiveness against both *S. aureus* and *E. coli* bacteria, they showed a higher diffusion of carvacrol through an agar gel inoculated with *S. aureus* than that with *E. coli*, resulting in a higher inhibition zone around the edible film area [217]. Moreover, the *E. coli* inhibition zone was larger for sodium caseinate films than for calcium caseinate films (Figure 8). This behavior was related to the fact that divalent calcium cations in calcium caseinate promote crosslinking with protein chains [214], retaining carvacrol more efficiently due to the more tortuous structure, which releases the active agent more slowly [217].

Imran et al. [219] developed sodium caseinate films that incorporated nisin, one of the most-used bacteriocins for food conservation, with high antilisterial and antistaphylococcal activity. Meanwhile, Calderón-Aguirre et al. [216] introduced nisin as well as antimicrobial substances produced by *Streptococcus infantarius* into glycerol-plasticized sodium caseinate films, and observed that caseinate films containing bacteriocins produced by *S. infantarius* showed higher antilisterial effectiveness in long-term refrigeration storage (around 2 months) than nisin-incorporated ones.

### 3.2. Keratin

Keratin is a protein found in mammalian hair, fur, wool, skin, hoofs, claws, and horns and in feathers of birds. This is an ancient material used for textile applications due to the early domestication of sheep and the use of the produced wool for such purposes. However, the need for non-contaminant sources for the design of sustainable bio-plastics has put the focus on keratin. Keratin extracted from such agricultural waste products as poor quality wool and chicken feathers has been used to produce films, fibers (electrospun fibers), and hydrogels and shown potential application as a scaffold for tissue engineering and tissue dressings and even as drug delivery systems [212,220,221,222]. Nevertheless, as is the case for most of the bio-based materials shown in this work, keratin is not antimicrobial by itself, so its functionalization or combination with antimicrobial agents is indispensable [223].

Having this in mind, Yu et al. [224] proposed the immobilization of quaternary ammonium moieties on a keratin-based substrate, thus turning keratin into an antimicrobial material for biomedical applications. The methodology consisted of the generation of thiols in wool keratin fibers (reduction of disulfide bonds using tris(2-carboxyethyl)phosphine hydrochloride) and then their reaction with the acrylate monomer [2-(acryloyloxy)ethyl]trimethylammonium chloride (2-AE) through click chemistry. In this way, a quaternary ammonium moiety was grafted onto reduced keratin fibers. The antimicrobial property of the obtained material and also of the untreated material was evaluated against *E. coli* bacteria using the agar diffusion plate test. Interestingly, the percentage of bacteria reduction obtained with the modified keratin was 94%, whereas for the untreated material there was no antibacterial effect. In this case, since the antimicrobial compound (the quaternary ammonium moiety) is covalently bonded to keratin, the antimicrobial activity is sustained in time and no leaking can occur. This interesting approach makes keratin-based materials applicable, for instance, as medical textiles.

Nayak et al. used a different approach by blending keratin with different polysaccharides (alginate, agar, and gellan) to obtain therapeutic porous dermal patches [225]. To impart antimicrobial activity, the obtained patches were coated with AgNPs. In particular, the antimicrobial activity of a keratin/agar patch was evaluated through the disk diffusion test against *S. aureus* and Gram-negative *Pseudomonas putida* bacteria and *A. niger* and *C. albicans* fungi pathogens. The results indicated the good antimicrobial activity of the patches against the tested pathogens, with a remarkable effect against *S. aureus*. Thus, the broad antimicrobial activity of the keratin-based obtained patches was confirmed.

### 3.3. Collagen

Collagen is the main structural protein found in the extracellular matrix of various connective animal tissues. The amino acids that compose collagen are wound together in a triple-helix, giving rise to elongated fibrils. The main role of collagen is both structural and functional, since it contributes to some processes of tissue repair [226]. In addition, by partial hydrolysis of collagen and destabilization of the triple-helix, it is possible to obtain the natural polymer called gelatin, which has also attracted much industrial interest. In sum, collagen possesses such properties as biocompatibility, biodegradability, and non-toxicity, which makes it suitable for applications involving wound healing and tissue regeneration. However, as is the case with the aforementioned bio-based polymers, this protein lacks the antimicrobial activity that is rather important for those kinds of applications. So, the modification/functionalization of collagen or its combination with antimicrobial agents is required.

Having this in mind, Balaure et al. [227] recently developed a collagen dressing containing orange essential oil functionalized ZnO nanoparticles (*d* = 20 nm) inserted into a three-dimensional (3D) matrix. As was described above, ZnO nanoparticles present antimicrobial activity, and their incorporation into collagen to impart biocidal properties is a strategy that has also been followed by other authors [228,229]. In this particular case, for the preparation of the dressing, suitable amounts of previously synthesized ZnO nanoparticles, collagen, and glutaraldehyde solution (crosslinker) were added so that collagen–ZnO gels were formed. For the antibacterial activity, collagen–ZnO gels (with three different ZnO contents) were placed in petri dishes and inoculated with *S. aureus* and *E. coli* bacteria strains. For the sake of comparison, antibiotic disks were used as a control. After 24 h of incubation, the diameters of the inhibition zone were measured and compared with the control disks. The results revealed that the collagen–ZnO wound dressings presented a remarkable antimicrobial activity against the *S. aureus* strain, as the growth inhibition zones were 11.5 mm and comparable to the diameter of the inhibition zones obtained for the control antibiotics. When analyzing antimicrobial activity against *E. coli*, the influence of the ZnO content was evidenced. Besides the antimicrobial activity, the developed collagen dressings showed great regenerative capacity. This combination of outstanding properties makes this bio-based sustainable material a potential candidate for wound healing applications.

Similarly, You et al. developed a metallic silver nanoparticles–collagen/chitosan hybrid scaffold to be used as a dressing for burn wounds [230]. Ideal dressings are required to present antimicrobial activity among other factors, such as keeping moisture in the wound, removing exudates, or even providing drugs that contribute to the healing. However, in the case of burn wounds, where a large amount of fluid is lost and bacterial infections occur, it can become necessary to have dressings with antimicrobial activity. For the scaffold’s preparation, bovine type-I collagen and chitosan were dissolved together in an acetic acid solution. In the next step, commercial AgNPs were added to the collagen–chitosan, poured in a plate and kept at 4 °C, frozen at −25 °C, and finally lyophilized to obtain the scaffolds. The antibacterial activity was evaluated against *S. aureus* and *E. coli* via the disk diffusion method, at which the zone of growth inhibition was measured after 24 h of incubation. An antimicrobial assay was performed for collagen and the hybrid collagen-based scaffolds, and, as expected, more activity was observed in the hybrid scaffold compared to the neat sample without AgNPs. Furthermore, the antimicrobial activity was improved as the amount of AgNPs in the scaffold increased. These bio-based hybrid scaffolds, besides their proven antimicrobial activity, exhibit anti-inflammatory properties that are also derived from the presence of AgNPs. As a conclusion, the authors postulated that the hybrid collagen-based dermal scaffolds could have application as a new antimicrobial dressing designed using non-toxic components.

Michalska-Sionkowska et al. [231] developed a collagen-based antimicrobial dressing that incorporated thymol as an essential oil to provide antimicrobial activity to the final material. As previously highlighted, it has been demonstrated that thymol shows antimicrobial activity against both Gram-positive and Gram-negative bacteria strains as well as against fungi and yeast [232,233]. For the preparation of the films, collagen obtained from rat tendons was dissolved in acetic acid. Then, different amounts of thymol were added to the collagen solution. For a better miscibility of the thymol in the collagen, a nonionic surfactant was also added to the solution. Finally, the solutions were poured, and, by the solvent evaporation method, collagen–thymol films were obtained. Since the main objective of the work was to develop an antimicrobial material against biofilm formation, the obtained films were tested by the agar diffusion method against *E. coli*, *P. aeruginosa*, *S. aureus*, *B. subtilis*, *E. aerogenes*, and *C. albicans* strains. The antimicrobial test revealed that the most sensitive micro-organism was *S. aureus* bacteria. This effect was even more significant with the increase in thymol content in collagen films. The growth of *E. coli* bacteria was also inhibited, although the dose needed was higher compared to that of *S. aureus.* Similar results were observed for *C. albicans*, *B. subtilis*, and *E. aerogenes*, but no inhibition was observed against *P. aeruginosa*. The biofilm formation for collagen–thymol and collagen control films was also evaluated against *S. aureus*. Through an SEM observation, it was concluded that the number of micro-colonies on the surface of the collagen-thymol film was smaller compared to that of the control film without the EO. Thus, collagen-based films were also effective in inhibiting biofilm formation.

## 4. Polyesters

Bio-based and biodegradable polyesters, such as poly(lactic acid) (PLA) and poly(hydroxyalkanoates) (PHAs), are currently the main drivers of the growth in the bioplastics market [2], as they have the potential to replace traditional polymers, such as poly(ethylene terephthalate) (PET).

As was already mentioned, antimicrobial systems based on biocompatible and biodegradable polymeric matrices have attracted interest for both biomedical and food-related applications. The main objective in developing antimicrobial polymeric systems is to lessen and subsequently prevent the micro-organisms from growing [234]. In this context, several strategies have been developed to provide materials with antimicrobial performance based on biopolyesters, most of which have been focused on adding additives with antimicrobial activity, including natural compounds (i.e., EOs), peptides (i.e., nisin), chelating agents, antibiotics, other polymers (i.e., chitosan), enzymes, and metals (zinc oxide, silver, etc.) [235]. Despite the wide range of antimicrobial agents that have been used during the last two decades for the development of biopolyester-based antibacterial materials, it seems that the route to introduce them is even more important than the agent itself in obtaining an effective antimicrobial activity at the surface of the material.

### 4.1. Poly(lactic acid) (PLA)

**Figure 9:**
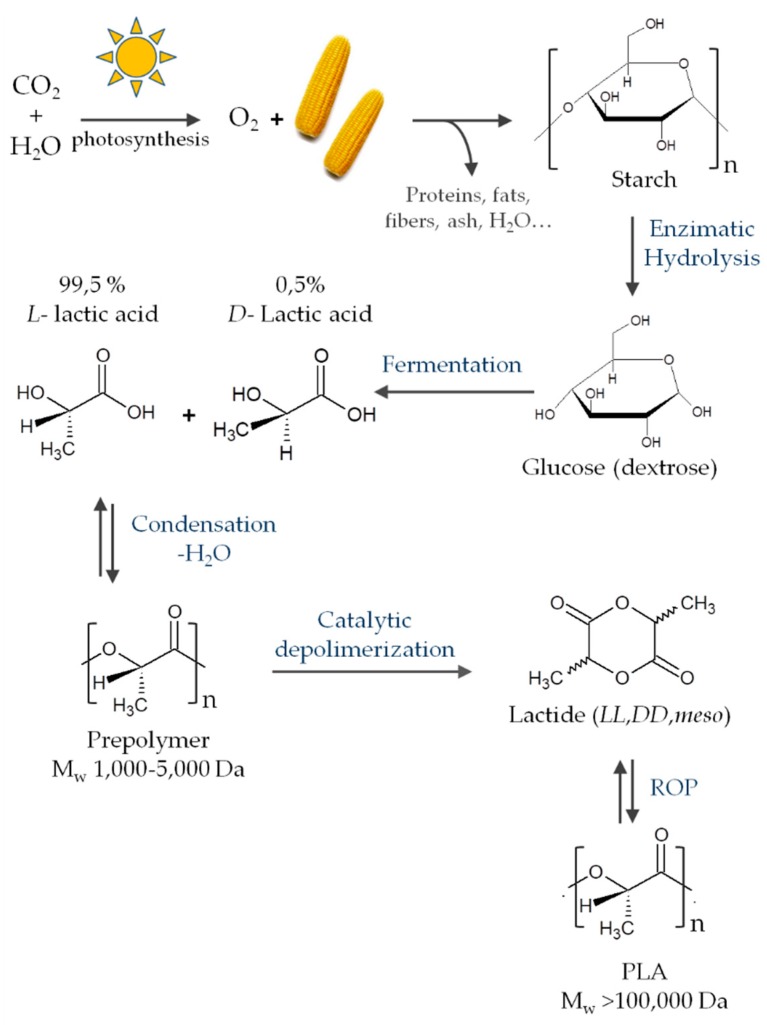
A schematic representation of high-molecular-weight PLA industrial production. Reproduced from [242].

Among all sustainable polymers, PLA is currently the most promising one to replace petroleum-derived polymers. Moreover, PLA is biocompatible and biodegradable, and this is why it is widely used in the biomedical field as well as in a wide range of commodity applications in other industrial sectors, such as the agricultural and food packaging sectors [236,237]. PLA is chemically synthesized starting with simple sugars obtained from agro-resources, such as corn, potatoes, cane, and beet, and fermented to the lactic acid monomer [236,237]. The most common route to produce PLA at the industrial level is the ring-opening polymerization (ROP) of the cyclic lactide dimer by condensation with metal catalysts (e.g., tin octoate) with the elimination of water at a high temperature (but less than 200 °C) [238,239] (Figure 9). Another important advantage that has allowed us to rapidly introduce PLA into the market is that PLA can be processed with the same processing technology that is already used at the industrial level for traditional petroleum-based thermoplastics to obtain films, molded pieces, and fibers, including melt blending, extrusion, injection molding, thermoforming, film forming, and electrospinning [236,240,241].

A laminated chitin PLA–PLA composite has been recently prepared by the hot-press method. Chitin powder was dispersed into a PLA matrix and further processed by a hot press to obtain the inner layer, where a neat PLA layer was also obtained by the hot press method [243]. It was observed that the incorporation of 5 wt% of chitin in the chitin–PLA layer in direct contact with a Mueller–Hinton agar medium, inoculated with *E. coli* bacteria, was enough to produce a clear inhibition zone. Besides this, antibacterial PLA-based compounds have been recently developed by adding chitosan nanoparticles in 0.5, 1, and 2 wt% into a PLA matrix by melt extrusion followed by a solvent casting process to obtain nanocomposite films. The obtained nanocomposites showed higher antibacterial effectiveness with increasing content of nanochitosan as well as higher antibacterial effectiveness against *L. monocytogenes* than against *E*. *coli* bacteria [243].

Imran et al. [219] introduced nisin into several bio-based and biodegradable matrices, including sodium caseinate, chitosan, and PLA. The antibacterial effectiveness was tested against *L. monocytogenes* and *S. aureus*. Only the PLA–nisin disc films showed effectiveness against both bacteria due to the PLA’s hydrophobic nature, which lead to a higher nisin retention ability.

EOs have been widely used as PLA antimicrobial additives for the development of active PLA-based materials with antibacterial activity, which have mainly been studied by the agar disk diffusion method to simulate the food-wrapping conditions [244,245,246]. Plasticized PLA composite films were developed by loading bimetallic silver–copper (Ag–Cu) nanoparticles and cinnamon essential oil into a polymer matrix via melt blending [247]. The obtained nanocomposites were used to pack contaminated chicken samples with Gram-negative *Campylobacter jejuni*, *L. monocytogenes*, and *S. Typhimurium*, and the degree of bacteria survival after 21 days under refrigerated storage conditions was measured. The bacterial survival of food-borne pathogens decreased significantly from 6.65 to 3.87 log CFU/g for *L. monocytogenes*, from 5.40 to 2.59 log CFU/g for *C. jejuni*, and from 5.52 to 2.42 log CFU/g for *S. typhimurium*, resulting in interesting materials for antimicrobial food packaging systems. However, when EOs are directly incorporated into thermoplastic polymeric matrices that should be processed at a high temperature, high amounts of EOs are lost during processing due to their high volatility. For instance, plasticized poly(lactic acid)–poly(hydroxyalcanoates) (PLA-PHA)-based blends were directly incorporated with 10 wt% of carvacrol and processed by melt extrusion [245,246]. Around 25% of the carvacrol was lost during thermal processing [245]. Nevertheless, due to the high amount added into the blend formulations as well as the high antibacterial activity of carvacrol, the films showed some antibacterial surface effectiveness against *S. aureus*, with the observation of a clear zone of growth inhibition around plasticized film samples, while no inhibition halo was observed in unplasticized samples. The increased mobility of the macromolecular chains, due to the plasticizer’s presence, promotes the diffusion of carvacrol from the polymeric matrix into the agar medium in a radial way, improving the antimicrobial effectiveness. However, no inhibition halo was observed in the agar plates inoculated with *E. coli* bacteria, even with plasticizer [246].

In this context, novel processing strategies other than thermal processing to obtain antimicrobial PLA systems have recently been developed to avoid the loss of volatile additives and to increase the antibacterial performance of the materials at the surface.

Supercritical impregnation has recently been proposed as an effective route to the introduction of volatile active compounds, such as EOs, into PLA matrices [248,249,250,251]. For instance, it has been recently used to incorporate thymol into PLA to develop materials with a wide range of applications [248,250]. Villegas et al. recently developed PLA films impregnated with cinnamaldehyde by supercritical impregnation with very effective antibacterial activity against *S. aureus* and *E. coli* bacteria [249].

Likewise, the production of ultrafine electrospun PLA fibers in the form of nonwoven materials has shown great potential in several fields, such as drug delivery, tissue engineering, filtration, catalytic processes, sensor development, and packaging. In fact, the electrospinning process has been suggested to be an effective way to produce nonwoven materials with active surfaces [251,252,253]. This technique allows for processing of the active substance with the PLA matrix at room temperature, avoiding the thermal degradation of the active substance. Moreover, during the preparation of a PLA material through the electrospinning technique, the high ionic strength as well as the rapid evaporation of the solvent induce the localization of the active compounds predominantly on the surface of the fibers [251,252]. For instance, cinnamaldehyde was incorporated into electrospun PLA-based materials by Lopez de Dicastillo et al. [251] following two approaches: direct incorporation into an electrospun PLA solution and supercritical impregnation of the electrospun PLA material. The authors observed that the materials into which cinnamaldehyde had been incorporated by supercritical impregnation showed a higher surface availability of the active compound, since those materials showed a higher release rate in fatty food simulants when compared to electrospun materials into which cinnamaldehyde had been incorporated during the electrospinning process.

Antimicrobial natural extracts with antibacterial activity can also be obtained from algae. For instance, natural extracts with some antimicrobial performance against *E. coli* have been obtained from *Durvillaea antarctica* algae and incorporated into a PLA matrix encapsulated in electrospun poly(vinyl alcohol) (PVA) fibers [253].

Nanostructured and aluminum-doped ZnO coatings were sputter-deposited onto an extruded PLA film by Valerini et al. [254] to functionalize its surface with antimicrobial activity. The materials with uniform surface coverage showed antibacterial effectiveness against *E. coli*. The authors concluded that the strong antibacterial effectiveness against Gram-negative bacteria is due to a polycrystalline structure, with the presence of cubic aluminum-doped zinc oxide (ZnAl_2_O_4_), ZnO, and aluminum oxide phases.

Antimicrobial electrospun PLA fibers have also been developed by adding silica nanoparticles functionalized with ZnO. The PLA-based materials’ antibacterial effectiveness against *E. coli* was found to be concentration-dependent and size-dependent. They required at least 0.8 wt% of functionalized zinc-oxide-doped silica NPs to produce a reduction in bacteria growth. Moreover, the authors obtained nanoparticles with an average diameter of 5–20 nm and observed that when the particle size was reduced, the surface reactivity of the nanoparticles was enhanced, leading to electrospun materials with better antimicrobial performance [255].

Another interesting technique for the modification of PLA film surfaces and the improvement of their functionalization is by plasma treatments [256,257,258]. For instance, Hu et al. [257] have recently coated a PLA film’s surface with nisin after modifying the film’s surface by means of cold plasma treatment (CPT). They studied the surface antimicrobial activity of the films against *L. monocytogenes* bacteria by the inhibition zone method. The authors found that the inhibition zone was only formed in the area where the incubated agar medium was in direct contact with the PLA–nisin-based films due to the poor water solubility of nisin. The antibacterial effectiveness of the PLA–nisin films was then determined quantitatively by means of the viable cell count method, and the total viable counts (TVCs) of *L. monocytogenes* decreased from 3.01 to 2.25 log_10_ (CFU/mL) by increasing the time of functionalization of the PLA’s surface by CPT from 15 to 60 s. These results suggest that increasing the CPT time positively affects the nisin adsorption capacity of the PLA’s surface as well as allowing for nisin to be released from the PLA’s surface when it is in an appropriate medium. Zhang et al. [258] treated electrospun materials with oxygen plasma to obtain a material with a hydrophilic surface that can be further functionalized with antimicrobial substances. Catechol possesses an essential role as an adhesive between interfaces; particularly, the synthetic catechol derivative dopamine methacrylamide monomer (DMA) possesses an exceptional adhesive property, which was used by these authors for surface modification in the development of biomedical devices. The electrospun PLA materials were modified with graphene oxide with DMA and the obtained nanocomposite showed excellent biocompatibility and exhibited antimicrobial properties against *S. aureus* and *E. coli* bacteria.

Munteanu et al. [259] prepared a nanocoating by encapsulating argan and clove oils into chitosan by coaxial electrospinning and then used it to functionalize electrospun PLA materials previously treated with CPT. The authors concluded that the electrospun coaxial encapsulation of clove and argan oils into chitosan led to enhanced antimicrobial activity against *E. coli*, *S. typhymurium*, and *L. monocytogenes*.

### 4.2. Poly(hydroxyalcanoates) (PHAs)

**Figure 10:**
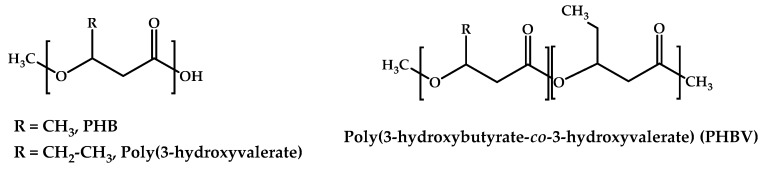
The chemical structure of most typical PHAs.

PHAs are a family of isotactic, semi-crystalline, and high-molecular-weight thermoplastic polyesters (Figure 10), which are biologically synthesized by controlled bacterial fermentation of a wide variety of both Gram-negative bacteria (i.e., those belonging to the genera *Azobacter, Alcaligenes*, *Bacillus*, and *Pseudomonas*) [260] and Gram-positive bacteria (i.e., those belonging to the genera *Nocardia*, *Rhodococcus*, and *Streptomyces*) [261]. In response to nutrient limitation (e.g., phosphorus, nitrogen, trace elements, or oxygen) and in the presence of an abundant source of carbon (e.g., glucose or sucrose) or lipids (e.g., vegetable oil or glycerine) [238], bacteria can accumulate up to 60–80% of their weight in PHA [261,262,263]. Therefore, PHAs are bio-based, biocompatible, and biodegradable, and exhibit thermal and mechanical properties that are comparable to those of other common polymers, such polystyrene (PS) and polypropylene (PP). In fact, the PHA family has been under development during the last few decades, the PHAs market is now emerging very fast, and the production capacity is predicted to quadruple in a few years [2].

Among the PHAs, poly(3-hydroxybutyrate) (PHB) is the most simple and common representative of PHAs. This is the reason why it is the most widely investigated for the development of PHA-based antimicrobial systems. From a processing point of view, the main drawback of PHB is its very low resistance to thermal degradation, since PHB has a melting temperature (around 170–180 °C) that is close to the degradation temperature (270 °C) [240]. Thus, although PHAs are still processed by melt compounding [264,265,266,267], the most recent studies on antimicrobial PHA systems are focused on the preparation of active PHA-based materials at room temperature [268,269,270,271].

PHB films with antibacterial properties have been developed by adding metal nanoparticles. In particular, PHB/AgNP nanocomposites are the most extensively developed group. PHB films have had AgNPs embedded into them by a two-step process consisting of helium plasma treatment followed by an immersion process in a silver nitrate solution [272]. Castro-Moyorga et al. [269] simultaneously biosynthesized AgNPs and PHB from the fermentation process of *Cupriavidus necator*. The obtained biopolymer and nanoparticles were used to produce bionanocomposite films with potential as active coatings for food packaging applications, since they showed antibacterial effectiveness against the food-borne pathogens *S. enterica* and *L. monocytogenes*.

**Figure 11:**
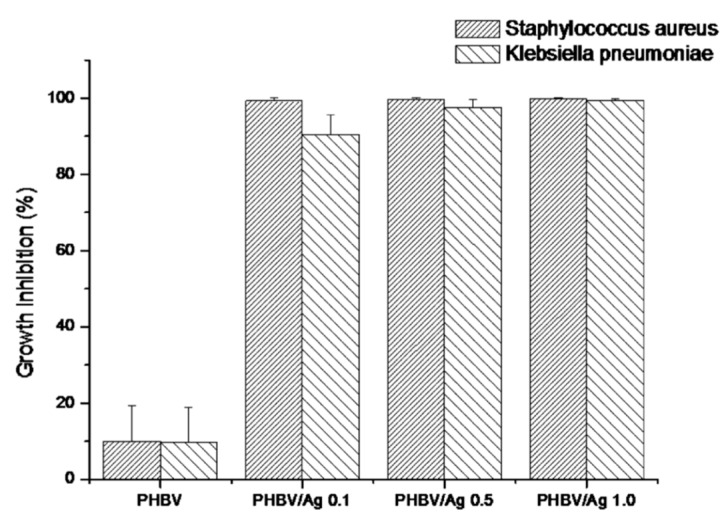
The growth inhibition of PHBV nanofibrous scaffolds with different amounts of silver against *S. aureus* and *Klebsiella pneumoniae*. Reprinted from [270].

**Figure 12:**
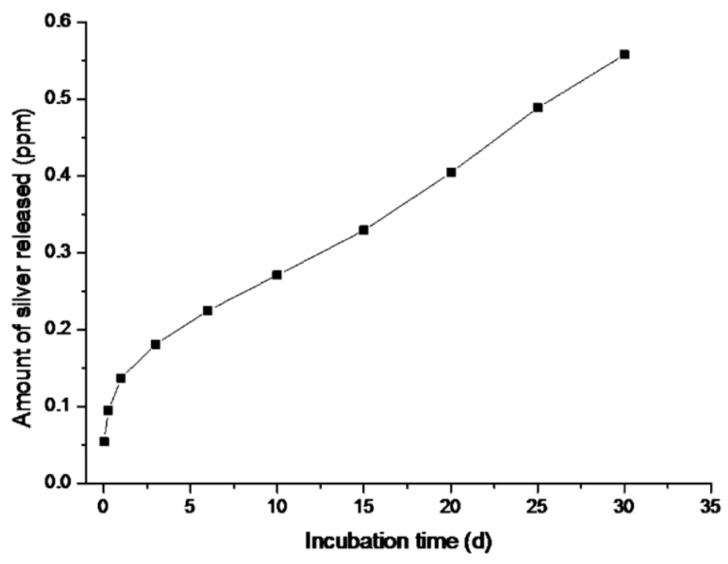
The amount of silver nanoparticles (AgNPs) released from PHBV/Ag 1.0 nanofibrous scaffolds as a function of the immersion time. Reprinted from [270].

Another PHA, poly(3-hydroxybutyrate-*co*-3-hydroxyvalerate) (PHBV), was loaded with less than 1 wt% of AgNPs and processed into an ultrafine electrospun fiber. It showed good in vitro cell compatibility and completely inhibited the proliferation of *S. aureus* as well as *K. pneumoniae* bacteria [270] (Figure 11). The amount of released AgNPs in distilled water increased with the incubation time, reaching a value of 0.557 ppm after 30 days (see Figure 12). The release is accelerated by the high surface area of nanofibrous scaffolds and the biodegradation of PHBV. Therefore, these materials may be good candidates for arthroplasty in regenerative medicine.

Zinc-oxide nanoparticles were also recently used in combination with PHA matrices. For instance, PHB was melt compounded with bacterial cellulose nanofibers to obtain nanocomposites, which were further modified by plasma treatment and coated with ZnO nanoparticles dispersed in alcohol using an ultrasonic spraying device [273]. The obtained nanocomposites completely inhibited the growth of *S. aureus* bacteria.

Naveen et al. [274] have prepared PHB electrospun nanofibers loaded with kanamycin sulphate, a water-soluble aminoglycoside antibiotic, for the development of nanoscaffolds for cell growth and antimicrobial devices. The electrospun scaffolds promote cell attachment, while the hydrophilic antibiotic confers antimicrobial performance on the electrospun surface as revealed by the obtained good zone of inhibition against *S. aureus* bacteria. The results showed that the electrospinning process does not affect the antibacterial activity of the kanamycin sulphate drug.

Poly(hexamethylene guanidine hydrochloride) granular polyethylene derivatives are of particular interest as additives in PHB-based materials processed by melt blending technologies, since they possess high thermal resistance [275]. Walczak et al. developed melt-blended PHB films enriched with poly(hexamethylene guanidine hydrochloride) granular polyethylene wax in 0.6–1 wt% with respect to the polymeric matrix. The active films were able to avoid bacterial biofilm formation by *S. aureus* on their surface [275]. Thus, the resulting materials are very interesting for biomedical as well as for food-related applications.

### 4.3. Poly(butylene succinate) (PBS)

**Figure 13:**
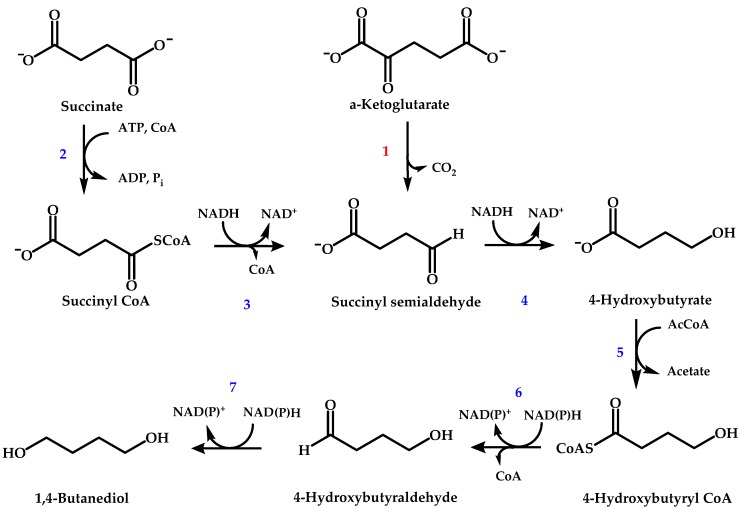
BDO biosynthetic pathways introduced into *E. coli* bacteria. Enzymes for each numbered step are as follows: (1) 2-oxoglutarate decarboxylase; (2) succinyl-coenzyme A (CoA) synthetase; (3) CoA-dependent succinate semialdehyde dehydrogenase; (4) 4-hydroxybutyrate dehydrogenase; (5) 4-hydroxybutyryl-CoA transferase; (6) 4-hydroxybutyryl-CoA reductase; (7) alcohol dehydrogenase. Steps 2 and 7 occur naturally in *E. coli*, whereas the others are encoded by introduced heterologous genes.

Poly(butylene succinate) is one of the most popular poly(alkylene dicarboxylate) polymers as it combines good properties with biodegradability, and can be produced by polycondensation of succinic acid and 1,4-butanediol (BDO) monomers, which are completely bio-based and obtained from refined biomass feedstock (from sugar-based feedstock by bacterial fermentation) [276,277,278]. In fact, many sources support the view that PBS is a material with the potential to replace polyolefins in the near future [279]. Nowadays, the easiest way and most-employed strategy to obtain succinic acid is from micro-organisms, such as fungi or bacteria, being the most intensively studied *Anaerobiospirillum succiniciproducens* [280] and *Actinobacillus succinogenes* [281] due to their ability to produce a relatively large amount of succinic acid. Interestingly, the use of glycerol as a carbon source substrate had been reported to lead to the best yields of succinic acid compared to other carbohydrates [280]. BDO can also be directly produced from biomass [277,282] or indirectly from bio-based succinic acid through its catalytic hydrogenation [283,284,285], being this latest route the most common. Figure 13 shows an innovative and promising pathway to obtain BDO in a one-step microbial fermentation process. Although, in most of the literature, PBS is produced using petroleum-based monomers, it is within the scope of the present work to include them, as nowadays both monomers that constitute PBS are bio-based, becoming more and more accessible, and slowly replacing the petroleum-based polymer. Therefore, PBS is expected to be completely included in the family of bio-based polymers in the near future as it is already produced by an industrial company [286,287].

Typically, PBS is employed in medical equipment and in the food industry, where sterile conditions together with biodegradability are usually required and the safety and shelf-life of food products must be ensured/improved. In these regards, antimicrobial properties are added to the material by the addition of antimicrobial agents. Those additives can be included in the polyester matrix to achieve the desired activity through the migration of the agent, or can be bounded chemically to its surface through functionalization, as stated for the above-reviewed polymers.

**Figure 14:**
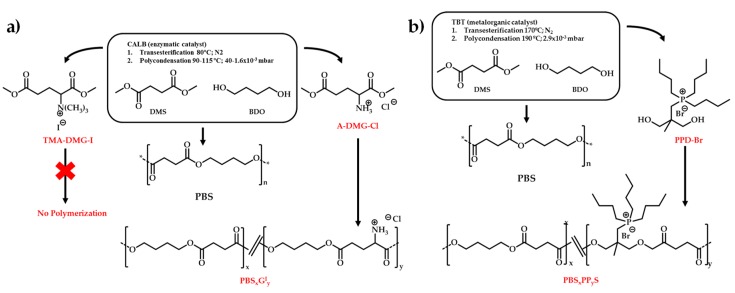
(**a**) An enzymatic synthesis route to a cationic PBS copolyester containing ammonium. (**b**) An organometallic catalyzed synthesis route to a cationic PBS copolyester containing phosphonium.

The first strategy consists in bounding specific functional groups or molecules to the polymer backbone. This polyester, in general, possesses a lack of available functional groups for further functionalization; therefore, most of the works introduce cationic groups that will provide antimicrobial activity into PBS chains by melt polycondensation to achieve cationic copolymers. Bautista et al. [288] copolymerized either 2,2-(dihydroxymethyl)propyl-tributylphosphonium bromide (PPD), 2-(N,N,N-trimethylammonium)dimethyl-glutarate iodide (TMA-DMG-I), or 2-ammonium dimethyl glutarate hydrochloride (A-DMG-CL) with succinic acid and BDO to obtain pendant quaternary phosphonium/ammonium groups on the copolymer chains (Figure 14). In order to overcome the drawbacks of the high temperatures needed for polycondensation reactions that can decompose cationic compounds, the authors successfully applied an enzymatic catalyst that was able to avoid metal residues and drive the reaction of ammonium-containing PBS under mild conditions (vacuum; 80–115 °C; 40–1.6 × 10^−3^ mbar). However, although at lower temperatures than is usual, the introduction of phosphonium groups was only possible by conventional polycondensation with a titanium catalyst (190 °C; 2.9 × 10^−3^ mbar). The antibacterial activity of the PBS copolyesters containing ammonium or phosphonium side groups at different concentrations was explored against *E. coli* and *S. aureus*. The phosphonium derivatives showed a strong antimicrobial effect with only 15 mol% of this cationic compound, while in the case of ammonium, the best results were obtained when 50 mol% of ammonium groups in the PBS were present.

Another way to obtain specific functional antimicrobial groups on PBS is the one followed by Wang et al. [289]. The authors modified the PBS surface by O_2_ or N_2_ plasma immersion ion implantation (PIII), a versatile technique that introduces chemical groups onto the samples depending on the gas employed. The authors evaluated the changes that occurred on the surfaces (chemistry, hydrophilicity) and their effects on the behavior in the presence of osteoblasts and bacteria. When the PBS surface was treated with O_2_, no difference was found with the control. However, when treated with N_2_, an antibacterial effect of 91.41% and 90.34% against *S. aureus* and *E. coli,* respectively, were obtained (from the amounts of active bacteria). This fact was associated with the presence of C=NH and C–NH_2_ groups that not only enhance the antimicrobial properties but also promoted osteoblast proliferation, differentiation, and mineralization, thus representing potential materials for implants.

Most of the reviewed literature on antimicrobial PBS is based on the incorporation of such ions as Cu or Ag, or essential oils that slowly migrate towards a product or media, into a PBS matrix. Research that considers PBS containing covalently bound antimicrobial components remains at a really early stage. However, the promising results that have been achieved to date will probably attract the attention of many researchers in the future.

**Figure 15:**
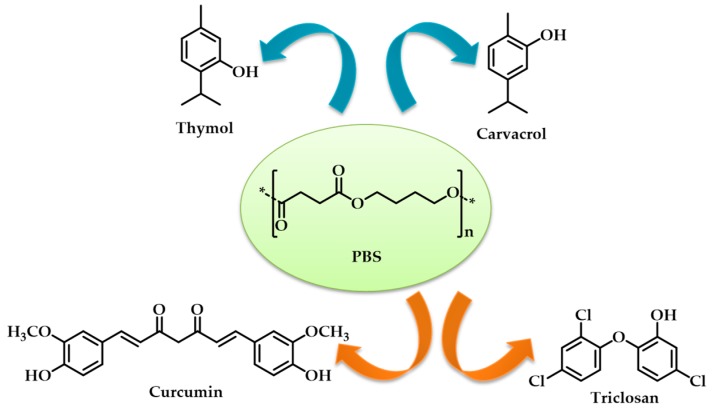
The chemical structure of PBS and agents employed to impart antimicrobial properties.

Generally, for the production of antimicrobial packaging, a common strategy is to use a bio-based EO and biopolymer (see Figure 15). Therefore, to produce antimicrobial PBS, Petchwattana et al. [290] incorporated thymol as an essential oil, which can be extracted from thyme (*Thymus vulgaris*), garlic (*Allium sativum*), and onions (*Allium cepa*) among other plants. As mentioned above, thymol possesses demonstrated antimicrobial efficiency for avoiding food spoilage and extending shelf-life [291]. Pure thymol has been demonstrated to possess antimicrobial efficacy against a broad range of micro-organisms, including Gram-positive *Listeria innocua* and *S. aureus* bacteria and *Saccharomyces cerevisiae* and *A. niger* fungi, with an MIC of 250 ppm for bacteria and mold and 125 ppm for yeast, retaining significant inhibition even under a microencapsulated condition [292]. In the same work, these authors also prepared PBS/thymol blown films, with antimicrobial food packaging applications as the target, containing 2, 4, 6, 8, and 10 wt% of thymol, and tested their antibacterial activity against *S. aureus* and *E. coli.* The release kinetic of the antimicrobial agent and its activity were evaluated. The MIC value was 10 and 6 wt% of thymol for *E. coli* and *S. aureus,* respectively. By incorporation of 10 wt% of thymol in PBS films, the antimicrobial agent release was effective over 15 days in all of the food simulants tested, while the maximum diffusivity was obtained in isooctane due to its identical polarity with thymol. In all of the tested systems, thymol migrated rapidly from the PBS matrix towards the food simulants, requiring 50–60 h to reach an equilibrium plateau in each case. Therefore, the authors claim that these materials are suitable for short-cycle food packaging applications, such as meat, vegetable, and fruit products.

A similar study conducted by Wiburanawong et al. [293] was focused on the addition of carvacrol to a PBS matrix as an antimicrobial agent for the preparation of food packaging. The prepared materials showed clear zones of inhibition of *S. aureus* and *E. coli* growth at 4 and 10 wt% of carvacrol; however, no release studies were conducted.

Jie et al. used the extract from *Scutellaria* root (*S. baicalensis*), a herb traditionally employed in Chinese medicine [294], to achieve a dual effect of dyeing and antimicrobial activity in PBS matrices. Natural pigments were mixed at 1, 3, 5, 7, and 9 wt% with PBS to achieve dyed films. The materials were tested against *S. aureus* and *E. coli* bacteria, achieving antimicrobial properties at the highest pigment load (9 wt%) without notably affecting the crystallization and thermal stability of PBS [295].

Although EOs are considered to be potential antimicrobial agents for PBS matrices, some authors claim that their strong odors might affect their acceptance on the market, and hence have tested alternative fillers as inorganic particles. The antimicrobial activity of ZnO-modified PBS films was tested against representative food spoilage bacteria (*S. aureus* and *E. coli*), and it was observed that a minimum content of 6 wt% was required for their growth inhibition with a slight increase in the inhibition zone diameter with the ZnO content. Release of Zn^2+^ ions from PBS was measured in distilled water, 3% acetic acid, and 10% ethanol food simulants and was found to have a strong dependence on ZnO concentration. During the tested 15 days, in distilled water and ethanol, Zn^2+^ released slowly, reaching a maximum at values lower than 10 ppm, while a fast release (similar to the PBS/EO systems discussed above) was observed on samples immersed in acetic acid, reaching a plateau value of 15 ppm after 50 h [296]. An applied study of a PBS film filled with ZnO NPs (10–30 nm in size), used as packaging to preserve and prolong the shelf-life of fresh-cut apples, was reported by Naknaen [297]. The author, packed, sealed, and stored freshly cut apple slices in PBS plastic bags with different contents of ZnO NPs (0, 2, 4, and 6 wt%) at 10 °C. After a three-day period (over a total of 18 days), quality parameters, such as color, weight loss, total acidity, and concentration of sugar, were determined. Additionally, a microbiological analysis was conducted at the end of the study over homogenized, filtered, and diluted samples in peptone water. Samples were plated onto agar, incubated, and then plate counted, showing a lower total amount of bacteria for samples containing ZnO NPs. By increasing the content of antimicrobial agent, a decrease in the bacteria population was achieved, reaching equal values for the samples containing 4 and 6 wt% ZnO NPs. Taking into account the most common regulation that limits the maximum count of aerobic micro-organisms to 6 log CFU/g at the expiration date [298,299], the shelf-life of the fresh-cut apple slices was enhanced by 8 days when packed in PBS containing 4–6 wt% of ZnO NPs (from 9 to 18 days).

**Figure 16:**
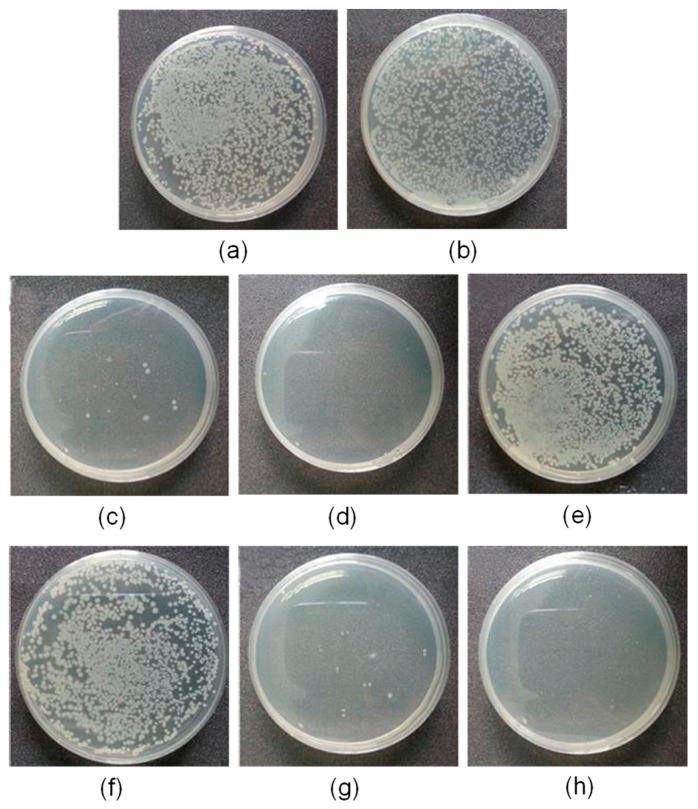
Photographs of *E. coli* (**a**–**d**) and *S. aureus* (**e**–**h**) colonies’ growth after incubation for 24 h with PBS (**a**,**e**), nano laponite (nLAP)-coated PBS (nLBC) (**b**,**f**), and Cu–nLAP-coated PBS (**c**,**g**) scaffolds with vancomycin as a positive control (**d**,**h**). Reproduced from [300].

**Figure 17:**
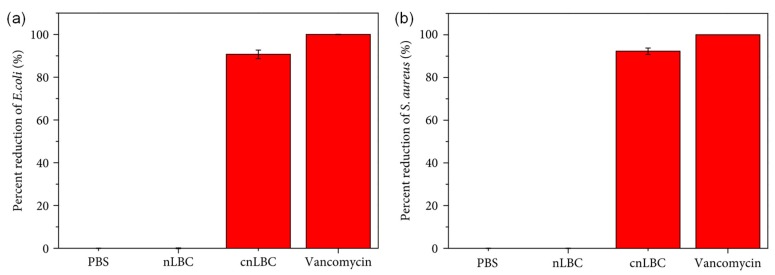
The percent reduction of *E. coli* (**a**) and *S. aureus* (**b**) bacteria on PBS, nLBC, and cnLBC scaffolds and vancomycin for 24 h. Reproduced from [300].

In the case of an application in the medical field, biodegradable polyesters are usually processed in the shape of fibers to find application as scaffolds. Tang et al. [300] coated PBS scaffolds, obtained using the salt leaching method, with copper-doped nano laponite (cnLAP). This material was to be applied in bone tissue engineering; therefore, the authors used nLAP to promote the osteogenic differentiation of human mesenchymal stem cells. Additionally, copper ions were expected to also promote bone regeneration and to inhibit infections. In fact, as is shown in Figure 16 and Figure 17, after the scaffolds were cultured for 24 h, only a reduction of bacteria (*E. coli* and *S. aureus*) was found in the coated samples containing copper (cnLBC), with values around 90% at the same time as no cytotoxicity occurred. Certainly, the incorporation of laponite improves the adhesion, proliferation, and differentiation of bone mesenchymal stem cells, which were ascribed to the release of Mg, Si, and Li ions from the coating on the scaffolds into the media. All of these facts make this system a good candidate for bone regeneration.

Tian et al. [301] introduced poly(vinyl pyrrolidone) capped with silver nanoparticles (PVP-capped AgNPs) into PBS electrospun materials to impart antimicrobial properties. PVP is often employed to stabilize AgNPs [302,303,304], and PVP-capped AgNPs have been reported to have better in vivo antimicrobial activity and less toxicity to mammalian cells [305] than other capped AgNPs [306]. Spherical capped AgNPs were successfully distributed and incorporated into the PBS electrospun fibers. The release of silver ions from the scaffolds was investigated in aqueous solution by an inductively coupled plasma spectrophotometer. After 2 weeks, the authors still detected silver ions released from the scaffolds due to the hydrophobic character of the PBS that hindered the permeation of water into the fibers and the diffusion of AgNPs from the fibers. Consequently, an antimicrobial capacity was confirmed against *S. aureus* and *E. coli*, obtaining an ability to inhibit bacterial growth in the long-term (more than 2 weeks).

Llorens et al. went further and developed a drug delivery scaffold constituted by electrospun poly(ethylene glycol) (PEG) and PBS blends. By coaxial electrospinning, different core–shell distributions were obtained, having either PEG or PBS in the outer or inner part (PEG-PBS and PBS-PEG core–shell distributions) in order to be compared with scaffolds obtained using a conventional setup (fibers obtained from a mixed solution of PEG and PBS). PEG and PBS solutions were loaded, respectively, with triclosan (polychlorophenoxy phenol at 1 w/v%), which is an antimicrobial and antifungal agent [307], and curcumin (0.5 w/v%), which is a natural phenol that seems to have beneficial effects on the treatment of several diseases [308,309]. The release profiles were studied for all of the scaffolds and revealed a high dependence on the media’s hydrophobicity and the structure of the fibers. The authors achieved a different release of curcumin and triclosan and claimed that the solubility of PEG in aqueous media led to a fast release of the antibacterial compound, while the non-aqueous solubility of the curcumin-loaded-PBS component will permit a sustained anticancer effect with time [310]. Additionally, antibacterial tests were performed against *E. coli* and *M. luteus*, determining the bacterial adhesion and growth onto triclosan-loaded scaffolds. All drug-loaded scaffolds prevented bacterial colonization effectively, while coaxial samples were found to be more susceptible to bacteria colonization (growth inhibition measurements) than their uniaxial electrospun counterparts. Thus, 60–40% of inhibition was obtained from the coaxial samples, without a significant influence on the core–shell structure, while 90–75% of inhibition resulted from the uniaxial electrospun scaffolds.

## 5. Polyurethanes Based on Renewable Oils

Natural oils and fats from vegetable oils (VOs) are the most important renewable industrial feedstock for sustainable chemistry and, indeed, in polymer science [311]. VOs are triacylglycerols formed from glycerol and three fatty acids, being the most common the saturated capric (C10), lauric (C12), myristic (C14), palmitic (C16), and stearic (C18) acids and unsaturated oleic (C18, with one carbon–carbon double bond) and linoleic (C18, with two or three carbon–carbon double bonds) acids. In general, VOs do not contain hydroxyl groups; therefore, they are often modified chemically to introduce hydroxyl groups into their structures. Interestingly, polymer scientists have found plant-oil-based polyols to be attractive thanks to their unique chemical structure that offers numerous options for modification. VOs are cheap, non-toxic, biodegradable, and, moreover, their polar character confers enhanced antibacterial properties to the resultant polymeric systems. Most utilized polyols from VOs are derived from soybean, sunflower, and cottonseed and, together with fatty acids, have been used for many years in the production of polyols employed in polyurethane and, to a lesser extent, in polyester synthesis [312,313].

**Figure 18:**
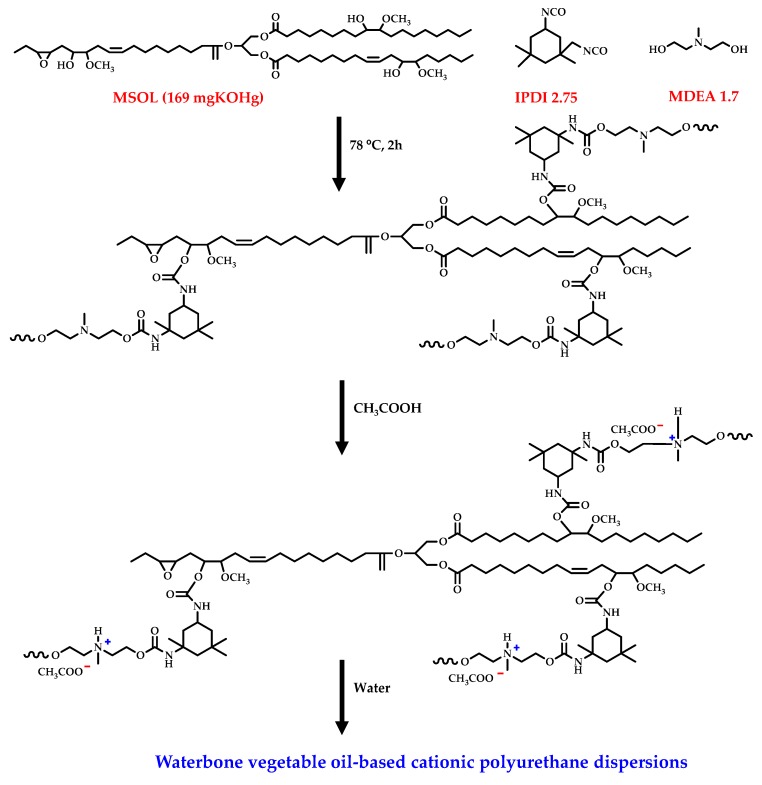
The synthesis route of the cationic soybean-oil-based polyurethane dispersions (PUDs). MSOL, Methoxylated soybean-oil polyol; IPDI, Isophorone diisocyanates; MDEA, *N*-methyldiethanolamine.

VOs have been employed for the synthesis of cationic polyurethane (PU) coatings with antimicrobial properties for application either in the biomedical or food packaging fields. Usually, cationic PUs are prepared by incorporating a tertiary amine diol or polyol treated with an acid that is able to bind microbes and disrupt their cell structure thanks to amino groups. Xia et al. [314] prepared cationic soybean-oil-based waterbone PU dispersions and coatings from five amino polyols (Figure 18), and examined the effect of their structure and hydroxyl functionality over the antimicrobial, mechanical, and thermal properties.

*N*-methyldiethanolamine (MDEA)- and *N*-ethyldiethanolamine (EDEA)-containing PUs provided the best antibacterial activity, which the authors associated with the smaller/shorter side chains attached to the nitrogen atoms that allow for better penetration of materials into cells. They observed a good antibacterial activity towards *L. monocytogenes* and *S. typhimurium* and against a Gram-negative structural mutant of *Salmonella minnesota* (R613) lacking a full outer membrane layer. Additionally, although the MDEA with a triethanolamine (TEA) residues structure had lower antibacterial activity than MDEA and EDEA (lower ammonium ion content), it provided the best balance of antimicrobial, thermal, and mechanical properties from all tested amino polyols thanks to its relatively high crosslink density. In a similar study, the same group [315] varied the molar ratio between hydroxyl and isocianate groups in the PU by varying the MDEA content. All formulations showed inhibitory activity against *S. typhimurium*, *L. monocytogenes*, and MRSA. Clearly, an increase in the ratio of ammonium cations enhanced the antibacterial properties. Additionally, they varied the crosslinking density of the materials by using methoxylated soybean polyols (MSOLs) with different numbers of hydroxyls. Lower crosslinking densities (a lower functionality of MSOLs) showed increased antimicrobial activities even though the concentration of quaternary ammonium was slightly lower than in their higher crosslinked counterparts. It seemed that a lower crosslinking density promoted the physical interaction with the target bacteria as the molecular mobility of the chains was higher, which helped to increase the antibacterial activity of the material.

Similarly, Liang et al. [316] employed castor oil, as a natural antimicrobial agent, and MDEA, as an ionic chain extender, to synthesize waterborne PUs. Samples were tested against *L. monocytogenes* and *V. parahaemolyticus* bacteria, being more effective by increasing either the MDEA content or the reduction of polyol functionality (a lower crosslinking density, higher mobility, and better interaction with bacteria).

Bakhshi et al. [317] functionalized intermediate tertiary amine soybean-oil-based polyols (TAPs) with ammonium salts by using either methyl iodine or benzyl chloride as alkylating agents instead of using an acid. These bio-based polyols containing quaternary ammonium salts were incorporated into polyurethanes using different diisocyanate monomers to obtain biocompatible and bactericidal coatings. When methyl iodine was used, the materials showed significant bacterial reduction (83–95%) against *E. coli* and *S. aureus* bacteria due to the higher amount of active groups in comparison to their benzyl-chloride-alkylated counterparts.

**Figure 19:**
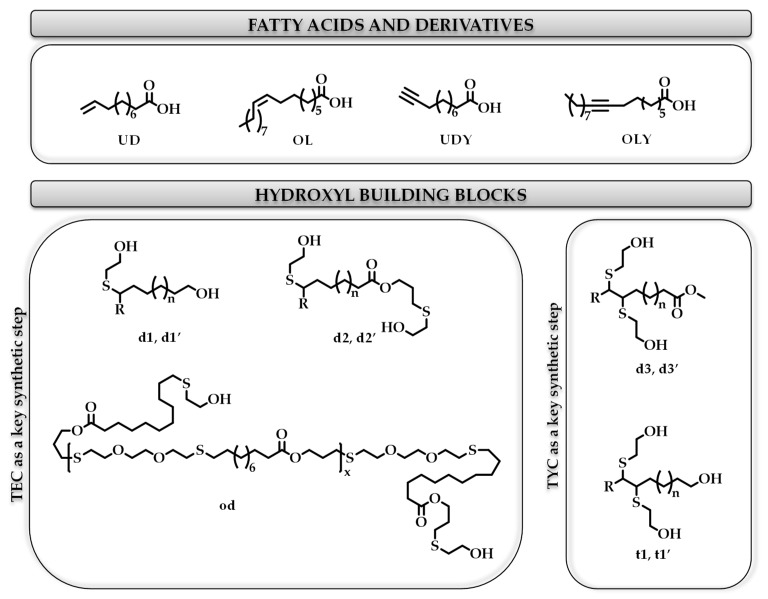
The chemical structures of 10-undecenoic (UD) acid and oleic (OL) acid, their alkyne derivatives (UDY and OLY), and the hydroxyl building blocks synthesized from them using either thiol-ene (TEC) or thiol-yne (TYC) as a key synthetic step (d, od, and t refer to diol, oligomeric diol, and triol, respectively, whereas 1, 2, and 3 refer to R = H and n = 6, and 1′, 2′, and 3′ to R = –(CH_2_)_7_–CH_3_ and n = 5).

**Figure 20:**
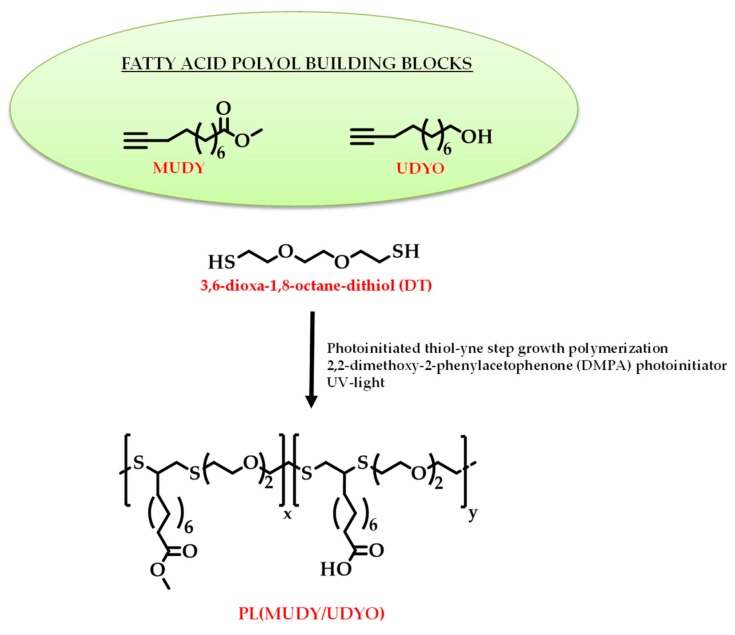
The chemical structures of reagents and the synthesis route employed to obtain methyl-ester-containing polyols from copolymerized methyl 10-undecynoate (MUDY) and 10-undecynyl alcohol (UDYO) fatty-acid-derived polyols. Varying MUDY/UDYO ratios allowed the researchers to obtain polyols with different hydroxyl contents for further reactions with diisocyanate to obtain polyurethanes (PUs).

Thiol-ene (TEC) and thiol-yne (TYC) couplings are the most commonly employed chemistries for the preparation of plant-oil-derived polyols (hydroxyl building blocks) (Figure 19) [318,319]. By applying thiol-yne coupling chemistry to alkyne-derivatized fatty acids from naturally occurring oleic and 10-undecenoic acids (mainly obtained from sunflower oil saponification and castor oil pyrolysis), bio-based methyl-ester-containing polyols for PU technology were obtained (Figure 20).

The synthesized polyols were used to prepare several PU formulations with enhanced surface hydrophilicity and antimicrobial properties after aminolysis with poly(propylene glycol) monoamine (Jeffamine^®^ M-600) and complexation with iodine. The antibacterial activity of these systems was tested against *P. aeruginosa*, *S. aureus*, and *C. albicans* [320], where effectiveness was only observed for Gram-positive bacteria and fungi but not for Gram-negative bacteria.

Algae oil and several di-acids from renewable sources (dimer acid, itaconic acid, maleic acid, and phthalic anhydride) were used to synthesize alkyd and polyesteramide polyols for PU coatings with anticorrosive and antibacterial properties [321]. Bactericidal properties were tested against *E. coli* and *S. aureus* by turbidimetry, showing that the uncoated polyols and the algae oil fatty amide (AOFA) and monoglyceride (MG) coatings had more bacterial attachment and less inhibition efficiency compared to algae-oil-modified PU coatings, since algae-oil-modified PU coatings have a higher percentage of oleic acid, which inhibits the bacterial growth.

In general, bio-based PU does not show antimicrobial activity and, in other polymeric families, the incorporation of uniformly dispersed metal or metal-oxide nanoparticles has been reported to result in nanomaterials with excellent antimicrobial activity [322]. These nanocomposites enhance the durability and efficacy of the antimicrobial effect through a controlled release of the fillers. The antimicrobial properties of *Mesua Ferrea L.* seed-oil-based hyperbranched and linear PU nanocomposites containing AgNPs were studied by Deka et al. [323]. The hyperbranched PU (HBPU) nanocomposite showed a better bactericidal effect over its linear counterpart (LPU) towards *S. aureus* and *E. coli* bacteria and against *C. albicans* yeast. The antibacterial activity was dose-dependent and, particularly at a high loading, the efficiency of the system was comparable to standard antibiotic and antifungal agents.

Moreover, Das et al. [324] reported the antibacterial activity of smart HBPU/Fe_3_O_4_ nanocomposites based on sunflower oil and with superparamagnetic-like behavior and shape-recovery effects. The authors concluded that the hyperbranched structure of the sunflower-oil-based HBPU matrix, as occurred with AgNPs, prevented agglomeration and led to a better dispersion of Fe_3_O_4_ NPs, resulting in better antibacterial performance against *S. aureus* and *K. pneumonia* with respect to bare Fe_3_O_4_. Both Fe_3_O_4_ nanomaterials and HBPU/Fe_3_O_4_ nanocomposites exhibited good antibacterial activity against infectious and biofilm-forming microbes.

Additionally, the same group demonstrated that the incorporation of multiwall carbon nanotubes (MWCNTs) decorated with Fe_3_O_4_ enhanced the antimicrobial capacity in comparison to non-nanohybrid HBPU nanocomposites filled either with Fe_3_O_4_ or MWCNTs [325]. Self-healable castor-oil-based polyurethane containing sulfur-nanoparticle-decorated reduced graphene oxide (SrGO), combining the potential of both antimicrobial agents (rGO and sulfur nanoparticles), was obtained by Thakur et al. [326] and further studied by Wu et al. [327]. A synergistic effect was obtained on the nanohybrid particles of SrGO, and, although a high dose of nanocomposite was needed, the HBPU–SrGO material showed an inhibitory effect on both Gram-positive and Gram-negative bacteria, enhancing the inhibitory effect of the neat matrix. Recently, Duarah et al. developed a bio-based hyperbranched PU from a starch-modified polyol filled with carbon dots and AgNPs (HPU/CD-Ag) as a material for rapid self-expandable stents. Importantly, HPU/CD-Ag nanohybrid membranes prevented biofilm formation against *E. coli* and *S. aureus* and bacterial adherence against *P. aeruginosa*, assessing the highest synergetic antibacterial activity in comparison to HPU/AgNP and HPU/CD nanocomposites [328].

A linseed-oil-based polyol was employed for PU synthesis, and nanocomposite films with 0.5–10 wt% of Biocera A^®^ commercial particles (composed of silver, zinc, magnesium, calcium phosphate, alumina, and silica, with a particle size of 3–4 µm) were obtained by the casting-evaporation technique. Interestingly, systems with and without commercial particles showed antibacterial performance against *E. coli*, *P. aeruginosa*, *S. aureus*, and *B. subtilis* and were not cytotoxic against the Murine fibroblast NIH 3T3 cell line. They suggested that these systems would be useful for wound dressing applications [329].

Boron-incorporated linseed oil polyols were also employed to obtain semi-inorganic vegetable-oil-based PUs [330]. Both polyols and their obtained PUs showed high antibacterial activity against *S. aureus*; however, although the polyols were completely inactive against *E. coli*, the PUs were found to be mildly to moderately active. Sharmin et al. [331] linked CuO NPs to a linseed oil polyol through an esterification reaction with copper (II) acetate (CuAc) in a one-pot, solvent-less process to obtain good antibacterial activity against *E. coli* and *S. aureus*, probably through membrane disruption and cell death. The authors proved as advantageous the small size of the particles, which enhanced the size of the contact surface area and improved the antibacterial action as the metal content increased. These materials may be useful for making a self-sterilizing biofilm that resists PU coatings and paints.

VOs and their derivatives have demonstrated huge potential as renewable feedstocks, receiving increasing interest from the research community for developing polyols and PUs from under-used sources of plant oils. However, there are still some challenges related to cost barriers, mainly due to the fact that they come from edible sources; so, their usage affects the cost of foodstuffs. Therefore, future efforts have to be concentrated towards the employment of non-edible seed oils, such as algae oils, for the preparation of bio-based polyols. Additionally, heterogeneity in terms of VO structure also represents a challenge and, additionally, new advances in bio-based isocyanates are expected to be made in the near future.

## 6. Concluding Remarks

There is a necessity to find new routes or alternatives to petroleum-based materials. The circular economy has to be reached in every corner of life, and, at the same time, we must be able to combat bacterial resistance to antibiotics. Thus, biopolymers are outstanding candidates to be modified or combined with an antimicrobial substance to obtain antimicrobial systems with application in several fields and in good alignment with the circular economy. This article describes some of the recent steps taken to reach these goals. However, more research and investment are needed to achieve fully sustainable materials with antimicrobial activity and effective substitutes for the existing ones. In this sense, nanotechnology as a method of reinforcement, nanoencapsulation, or nanostructuration has shown that it can help us to achieve our goals.
